# Supplementary material for: Noncoding variants alter GATA2 expression in rhombomere 4 motor neurons and cause dominant hereditary congenital facial paresis
Source: Nat Genet. 2023 Jun 29;55(7):1149–63. doi: 10.1038/s41588-023-01424-9 (PMC10335940; doi:10.1038/s41588-023-01424-9)
Supplement: Supplementary file 1 — Supplementary Clinical note, Tables 1–4, Methods and References. [file 41588_2023_1424_MOESM1_ESM.pdf]

nature genetics

Article https://doi.org/10.1038/s41588-023-01424-9

Noncoding variants alter GATA2 expression in rhombomere 4 motor neurons and cause dominant hereditary congenital facial paresis

In the format provided by the authors and unedited

Supplementary Clinical Note

Among the 58 CFP pedigrees analyzed, HCFP1 SNVs or duplications were identified in 11 of 12 pedigrees with vertical transmission (92%, one *de novo*), and in three of seven simplex cases with documented bilateral facial weakness (43%, one *de novo*), but not in an additional 31 simplex cases (primarily with unilateral facial weakness) or in eight pedigrees with horizontal or unknown transmission. Of note, among the mutation-positive individuals who underwent WGS from either cohort, no additional CNVs or rare SNVs were identified within the minimum duplicated region.

Of the three mutation-positive simplex cases, one harbored a *de novo* variant (Fam5:II-1), the second was the offspring of an examined and clinically unaffected mutation-positive father (Fam7:I-1), and the third was the offspring of the unexamined but reportedly unaffected mutation-positive parent and grandparent (Fam6:III-1). As noted above, all three of these individuals had bilateral facial weakness. By contrast, among the 31 mutation-negative simplex cases we screened, only four (12.5%) were reported to have bilateral facial weakness.

For the 14 pedigrees harboring HCFP1 variants, Supplemental Table 1 provides data for each family's race/ethnicity, geographic location, and genetic variant, as well as each participating family member's age, sex, exam site, CFP phenotype, and summary of brain MRI, EMB/NC, and audiology testing if obtained, and any other notable findings. Figure 2 provides photographs highlighting the variable degree of facial weakness and corresponding facial nerve hypoplasia seen by magnetic resonance imaging.

The symptomatic mutation-positive research participants manifest congenital facial weakness as life-long difficulty closing their eye(s), raising their eyebrow(s), wrinkling their forehead, sucking through a straw, and/or smiling and making other facial expressions (Figure 2). These symptoms led to smile and eyelid surgery in two individuals and isolated eyelid surgery in three additional individuals. As one might predict for a disorder resulting from altered gene regulation, the HCFP1 variants result in a variable, primarily bilateral CFP less severe than that typically found in Moebius syndrome or HCFP3[1,2].

We focused on 21 members of cohort 1 to better characterize the HCPF1 congenital facial weakness phenotype and identify any associated signs and symptoms. Of these, 12 mutation-positive participants were examined at the NIH Clinical Center (eight members of Fam1, two members of Fam3, and two members of Fam9) under a dedicated protocol titled 'Study on Moebius syndrome and other congenital facial weakness disorders' (ClinicalTrials.gov identifier: NCT02055248). Nine additional participants from cohort 1 had facial movements evaluated by a facial animation specialist by video recording, photographs, and/or in-person examinations. When possible, standardized photographs and videos of different facial expressions were obtained including visualization of the face: 1) at rest, 2) with elevated eyebrows, 3) with gentle closing of the eyes, 4) with tight closing of the eyes, 5) with best smile, 6) with biggest smile, 7) with puckered lips, 8) with showing the bottom teeth. On note, we were unable to examine or review videos of the remaining mutation-positive participants, including the three members of Family 6, two of whom were reported to be unaffected.

Among these 21 mutation-positive individuals evaluated more comprehensively, four had not considered themselves affected. On review of their facial movements (blinded to genotype), three were diagnosed with mild facial weakness (Fam3:II-2, Fam8:II-1, Fam8:II-2) while one, who harbored the variant present in gnomAD, had no visible weakness (Fam7:I-1).

Among these 20 mutation-positive individuals with detectable facial weakness, 16/20 (80%) had bilateral asymmetrical facial weakness; eight were weaker on the right compared to the left side, seven were weaker on the left compared to the right side, and one was mixed. Four individuals had unilateral facial weakness (three left-sided and one right-sided). In addition, among the 20 individuals, 11 had upper greater than lower facial weakness, six had equal upper and lower facial weakness, one was mixed, and only two had lower greater than upper facial weakness.

Five affected individuals underwent prospective magnetic resonance imaging (MRI) of the brain and cranial nerves at the NIH Clinical Center while retrospective clinical scans from two additional individuals were reviewed. In all scans, the facial nerves were thin or not visible, while the brain, other cranial nerves, and facial and extraocular muscles appeared normal (Figure 2, Supplementary Table 1).

Seven affected individuals underwent electrophysiological testing at the NIH Clinical Center. Nerve conduction studies revealed normal to low amplitudes and normal latencies in the facial nerves in the absence of other peripheral nerve or muscle defects, consistent with facial nerve neuropathy. Needle EMG of orbicularis oculi, orbicularis oris and/or mentalis muscles revealed normal or reduced insertional activity without abnormal spontaneous activity or muscle membrane irritability. In some cases, motor units were large amplitude, long duration, and polyphasic with reduced recruitment pattern and in other cases they were absent, consistent with a chronic neurogenic process. On activation, interference patterns were reduced. Blink studies showed normal latencies, prolonged latencies, or absent R1 and R2 waveforms (Supplementary Tables 1, 2).

Eight affected individuals underwent auditory testing at the NIH Clinical Center (Supplementary Table 1). All individuals had normal peripheral hearing sensitivity with the exception of one 47-year-old female (Fam1:III-2) who had mild, bilateral high-frequency hearing loss that was within the 95th percentile for age-related audiometric thresholds, and one two-year-old whose age precluded a successful behavioral audiometric evaluation. Auditory brainstem response (ABR) studies were normal in all seven patients who received testing, indicating functional integrity of the auditory nerves and the auditory brainstem pathways. Acoustic stapedial reflex (ASR) threshold testing was conducted in all patients except for the previously mentioned 2-year-old. Acoustic stapedial reflex thresholds were either elevated or absent in six of the seven patients tested (85.7%), with only one patient exhibiting a normal ASR threshold pattern. In the presence of facial paralysis/paresis, abnormal ASR is consistent with hypoplasia or absence of the facial nucleus and nerve proximal to the origin of the stapedial branch of CN 7. Notably, while *cRE1*$^{+/dup}$ embryos generate an excess of IEEs at the expense of FBMNs, it is not known if these cells survive or establish correct connections, and clinical testing is not sufficiently sensitive to detect enhanced IEE function. Therefore, it remains unclear if excess IEEs in humans or mice, if present, have clinical consequences.

Some affected participants had additional signs or symptoms, none of which segregated with HCFP. Full-scale intellectual quotients were generated for eight individuals and ranged from 108-123. Moderate intellectual disability and learning disabilities were each reported once in untested individuals. Four carried a diagnosis of attention deficit hyperactivity disorders (ADHD), five reported anxiety, and four reported depression or bipolar disorder. A subset of affected individuals had craniofacial dysmorphisms, including flat midface with absent nasolabial folds, upturned nasal tip, slit-like nares, micro/retrognathia, and vaulted palate. Three subjects had preauricular tags or pits, but no other ear malformations. One mutation-positive participant had Duane retraction syndrome, one had nonspecific unilateral restricted eye movements, one had saccadic smooth pursuit, and two had comitant strabismus. One had tongue

deviation to the right and one had uvula deviation to the left. Otherwise, tongue shape and protrusion were normal and no subjects expressed abnormal taste or lacrimation. Six affected individuals had mild scoliosis and/or kyphosis, four had pes planovalgus, and three had generalized hypermobility. Additional findings resembling hereditary connective tissue disorders were reported in single cases across different families, including mitral valve prolapse, pneumothorax, umbilical hernia, and keloids. In Family 1, two subjects had enamel hypoplasia of primary/deciduous teeth, while four had psoriatic/eczematous dermatitis. One individual in Family 7 had an ectopic kidney and bilateral duplicated ureters and three had histories of kidney stones. Renal ultrasound in five individuals revealed early medullary nephrocalcinosis in one and was normal in four. None had cardiac anomalies, and echocardiograms were normal in the six individuals tested. None had limb weakness or sensory complaints.

Notably, we did not identify any phenotypic features that distinguished the individuals harboring SNVs from one another or from those harboring duplications.

Supplementary Table 1. Clinical characteristics of HCFP1 mutation-positive individuals

Family Race/ Ethnicity, Location,[a] Genetic variant (GRCh37 chr3)	Gen-Ind, age at last exam, Sex[b]	Exam site[c]	CFP Phenotype[d]	Brain MRI[e]	EMG/NCS[f]	Audiology:[g] Hearing ABR ASR	Other findings
Family 1 White, N-H[h] MEA[i] *g.128,161,355-128,192,750 dup*	II-1 80, M	NIH	B, Rt>Lt, eyelid surgeries	na[j]	na	na	Urolithiasis, kyphosis, multiple basal cell cancers
	III-2 47, F	NIH	B, Lt>Rt, U>L, s/p facial sling and eyelid surgery	CNVII: Rt1, Lt0	CN VII neuropathy	Bilateral mild, age-appropriate, high freq HL nl[k] Abnormal	Anxiety, mild leg length discrepancy, asymmetric foot alignment
	III-3 43, M	NIH	B, Rt>Lt, U>L, s/p eyelid surgery	CNVII: R2, Lt0-1	CN VII neuropathy	nl nl Abnormal	Co-activation of right platysma with orbicularis oris, deciduous teeth enamel hypoplasia, focal dystonia, urolithiasis, xerosis
	III-5 36, F	NIH	B, Rt>Lt, U>L	na	na	na	Benign positional vertigo, constipation
	IV-1 8, M	NIH	B, Lt>Rt, L>U	na	na	na	ADHD, anxiety, Rt preauricular tags, constipation, normal renal US
	IV-2 5, F	NIH	B, Rt>Lt, U>L	na	na	na	Feeding difficulties, speech tx, GERD, constipation, eczema, hypermobility
	IV-4 11, M	NIH	B, Rt>Lt, U>L	CNVII: Rt1, Lt2	CN VII neuropathy	nl nl Abnormal	Deciduous teeth enamel hypoplasia, early medullary nephrocalcinosis, kyphoscoliosis, pes planovalgus, pityriasis alba
	IV-5 10, F	NIH	B, Rt>Lt, U>L	na	Borderline	nl nl Abnormal	Tourette syndrome, xerosis, pityriasis alba
Family 2 White, N-H, MEA *g.128,174,929-128,194,582 dup de novo*	II-2 57, F	Limited photos	B, Rt>Lt	na	na	na	Anxiety, depression, complex partial seizures
	III-2 14, M	Limited photos	B	CNVII: Rt0, Lt0	na	na	ADHD, anxiety, chronic nausea
Family 3 Multi-race, Hispanic *g.128,178,260 G>C*	II-2 65, F	Video	Lt, mild, self-reported unaffected	na	na	na	None
	III-2 36, F	NIH	B Rt U>L, Lt L>U	CNVII: Rt2, Lt2	CN VII neuropathy	nl nl nl	Bipolar, Rt preauricular tag, micro/retrognathia, high palate, severe malocclusion, enamel defects/heavily restored dentition, Rt tongue deviation, mitral valve regurgitation, urolithiasis, mild thoracic scoliosis, pes planovalgus
	III-3 31, M	Photos	B, Rt>Lt	na	na	na	Moderate intellectual disability, depression, saccadic smooth pursuit, micrognathia
	III-5 23, M	NIH	B, Lt>Rt	na	CN VII neuropathy	nl nl Abnormal	ADHD, depression, preauricular pit, vaulted palate, mild scoliosis, pes planus, keloid
	IV-1 6wk, M	Video	Lt, L>U	na	na	na	Micro/retrognathia
Family 4 White, N-H MEA *g.128,178,261 G>A*	IV-2 71, F	Report	B, incomplete, s/p eyelid surgeries	na	na	na	Micrognathia, orthognathic surgeries
	V-2 45, F	Report	B, incomplete	na	na	na	na
Family 5 White, N-H MEA *g.128,178,262 T>C de novo*	II-1 13, F	Video	B, Lt>Rt, eyelid and smile surgeries	CNVII: Rt1, Lt1	na	na	na
Family 6 White, N-H Lebanon *g.128,178,295 G>T*	I-2 Elderly, F	Report	Unaffected by report	na	na	na	na
	II-2 27, F	Report	Unaffected by report	na	na	na	na
	III-1 2, M	Report	Rt, affected by report	na	na	na	Intermittent exotropia, speech tx, frequent ear infections
Family 7 Multi-race, Hispanic	I-1 39, M	Video	No weakness, self-reported unaffected	na	na	na	na

g.128,178,297 A>G	II-2<br>9, F	Video	B, Lt>Rt	na	na	na	Learning disabilities, ADD, anxiety, mild hypotonia, R ear helix deficiency, high palate, mild neck webbing, ectopic kidney, duplicated ureters, supraumbilical hernia, pes planus
Family 8<br>White, N-H<br>Australia<br>*g.128,178,297 A>G*	I-1<br>43, M	Video	Lt, U>L, mild, self-reported unaffected	na	na	na	None
	II-1<br>10, M	Video	B, Lt>Rt, U>L	na	na	na	Lt non-accommodative esotropia
	II-2<br>7, F	Video	Rt, U>L, mild, self-reported unaffected	na	na	na	Duane retraction syndrome type 1
Family 9<br>White, N-H<br>MEA<br>*g.128,178,298 G>A*	IV-1<br>27, M	NIH	B, Lt>Rt, U>L	CNVII: Rt0, Lt2	CN VII neuropathy	nl<br>nl<br>na	Incomplete abduction and loss of elevation OD, up-shoot at extreme adduction OS, recurrent spontaneous pneumothorax, hypermobility, mild thoracic scoliosis, pes planovalgus
	V-1<br>2, M	NIH	B, Rt>Lt	na	na	na	Joint hypermobility
Family 10<br>White, N-H<br>Pakistan<br>*g.128,165,668-128,188,763 dup*	I-1<br>38, M	Report	L, Lt	Nl, report	na	na	na
	II-1<br>20y, M	Report	B, L; Lt>Rt	na	na	na	na
	II-2	Report	B, L	na	na	na	Glasses for myopia
Family 11<br>White, N-H<br>Brazil<br>*g.128,161,298-128,187.620 dup*	I-2<br>na, F	Report	B, U&L	na	na	na	Epicanthal folds
	II-2<br>na, F	Report	B, U&L	na	na	na	Epicanthal folds, micrognathia, fibromyalgia
	II-3<br>na, M	Report	B, U&L	na	na	na	Epicanthal folds, micrognathia
	III-1<br>31, F	Limited photos	B, U&L	na	CN VII neuropathy	na	Epicanthal folds, complex migraines
	IV-1<br>2m, M	Limited photos	B, U&L	na	na	na	Epicanthal folds
Family 12<br>White, Hispanic<br>Spain<br>*g.128,124,679-128,195,568 dup*	I-2<br>52, F	Limited photos	Lt, mild	na	na	na	na
	II-1<br>18, F	Limited photos	Lt	na	CN VII neuropathy	na	B hypermetropia, L>R
	II-2<br>13, F	Limited photos	B, Rt>Lt	na	na	na	B hypermetropia
	II-3<br>11, M	Limited photos	B, Lt>Rt	na	na	na	na
Family 13<br>White, N-H<br>France<br>*g.128,178,262 T>G*	I-1<br>50, M	Report	B	na	na	na	Type 2 diabetes mellitus
	II-1<br>9, F	Photos	B, Rt>Lt	na	na	na	Myopia, micrognathia, small tongue, lactose intolerance
	IV-1<br>7, F	Photos	B,<br>Rt U>L<br>Lt L>U	na	na	na	High palate, low-set R ear, slight developmental delay & learning disabilities
Family 14<br>White, N-H<br>Netherlands<br>*g.128,178,298 G>A*	I-2<br>87, F	Photos	No weakness, self-reported unaffected	na	nl	na	na
	II-1<br>62, M	Photos	R, U, mild, self-reported unaffected	na	na	na	na
	II-3<br>60, M	Photos	B, Rt>Lt	na	CN VII neuropathy	na	Mild epiphora
	III-1<br>29, M	Photos	B<br>U, Lt>Rt,<br>L, Lt=Rt	na	na	na	na
	III-3<br>22, M	Photos	B, U&L	na	na	na	Perinatal asphyxia due to nuchal cord, no resulting developmental or motor concerns
	III-4<br>35, F	Photos	B	na	na	na	na
	III-5<br>34, M	Photos	B<br>smile surgery	na	na	na	Epiphora
	IV-1<br>11m, M	Photos	B	na	na	na	Syndactyly of 2nd and 3rd Rt toe

[a]Location USA unless otherwise stated; [b]Generation and individual position in pedigree (Gen-Ind), Male (M). Female (F). [c]Exam Site: evaluated at NIH (NIH); facial movements evaluated by review of video recordings (Video, and a subset of those with video review were also evaluated in person by a member of the consortium) or photos (Photo); exam data were gathered from verbal or written report (Report). Those bolded are the 21 for whom details are summarized in Supplementary Clinical Note. [d]Congenital facial palsy phenotype: bilateral (B), left (Lt), right (Rt), upper (U) or lower (L) part of the face. [e]The only brain MRI abnormality found was

the diameter of facial nerve (CN VII) and denoted as mildly hypoplastic (2), markedly hypoplastic (1), or absent by MRI (0). [f]Facial nerve neuropathy (CN VII NP); For detailed results of electromyography and nerve conductions studies (EMG/NCS) refer to Supplementary Table 2. [g]Audiology testing included pure thresholds, auditory brainstem response (ABR), and acoustic stapedial reflexes (ASR). [h]N-H: non-Hispanic, [i]MEA: mixed European ancestry, [j]na: no data available.[k]nl: normal.

Supplementary Table 2. Electrophysiology Data

			Normal adult Values[3]	Fam1:III-2	Fam1:III-3	Fam1:IV-4	Fam1:IV-5	Fam3:III-2	Fam3:III-5	Fam9:IV-1
**Age**				47F	43M	11M	10F	36	23	27
**Sensory limb**	**Median**	Amp (µV)	>15	-	32	-	-	48	47	41
		CV (m/s)	>50	-	57	-	-	70	64	61
	**Sural**	Amp (µV)	>6	-	11	-	-	10	14	8
		CV (m/s)	>40	-	40	-	-	39	54	54
**Motor limb**	**Median**	Amp (mV)	>4.5	-	6.6	-	-	6.1	6	14.5
		CV (m/s)	>50	-	50	-	-	56	61	57
		F-Wave (ms)	<31.0	-		-	-	25.1	27.2	28.8
	**Peroneal**	Amp (mV)	>2.5	-	8.6 (L)	-	-	5.6	4.9	4.6
		CV (m/s)	>40	-	51 (L)	-	-	46	50	49
		F-Wave (ms)	<56.0	-	55 (L)	-	-	45.7	45.7	54
**EMG - limb**				-	Normal	-	-	Normal	Normal	-
**Motor face**	**Facial R**	OOC Amp (mV)	≥ 1.0	**0.1**	**0.1**	**0.5**	1	2.7	1.2	**0.1**
		OOC Lat (ms)	≤3.2	2.3	2.7	2.9	2.5	1.2	2.6	**5.2**
		OOR Amp (mV)	≥ 1.0	**0.5**	1.4	2.4	2.6	1.5	1.1	1.5
		OOR Lat (ms)	≤4.2	3.5	1.8	2	1.8	1.6	3.4	4.4
	**Facial L**	OOC Amp (mV)	≥ 1.0	**0.1**	**0.3**	**0.8**	1	2.4	**0.6**	**>0.1**
		OOC Lat (ms)	≤3.2	2.3	2.6	3	2.9	1.9	1.5	**3.9**
		OOR Ampl (mV)	≥ 1.0	**0.4**	1.6	2.9	1.7	1.3	**0.7**	**0.2**
		OOR Lat (ms)	≤4.2	2.8	1.8	2.8	1.6	1.3	1.4	3.9
**Blink Reflex (ms)**		R1 Lat ipsi (R)	≤13	**22.8**	**NR**	10.1	8.6	10.2	11.2	**NR**
		R1 Lat ipsi (L)	≤13	**16.8**	**NR**	10.8	10.7	11.6	11.3	**NR**
		R2 Lat ipsi (R)	≤41	**48.9**	**NR**	35.6	32	37.9	33	**NR**
		R2 Lat ipsi (L)	≤41	**44**	**NR**	31.6	40.6	38.9	**43.4**	**NR**
		R2 Lat contra (R)	≤44	39.3	**NR**	31.9	30.9	35.2	41.7	**NR**
		R2 Lat contra (L)	≤44	**51.2**	**NR**	34.8	42.9	38.7	40.8	**NR**
		R2 Lat Diff (R)		9.6	**NR**	3.7	1.1	2.7	8.7	**NR**
		R2 Lat Diff (L)		7.2	**NR**	3.2	2.3	0.2	2.6	**NR**
**EMG - face**		OOC (upper face)		-	**No motor units**	-	-	**Neurogenic**	-	**Neurogenic**
		Ment (lower face)		-	**Neurogenic**	-	-	**Normal**	-	**Neurogenic**
**Diagnosis**				**CN VII Neuropathy**	**CN VII Neuropathy**	**CN VII Neuropathy**	**Borderline**	**CN VII Neuropathy**	**CN VII Neuropathy**	**CN VII Neuropathy**

CV = conduction velocity, OOC = Obicularis occuli muscle, OOR = orbicularis oris muscle, Ment = Mentalis muscle, Amp = amplitude, Lat = latency, ipsi = ipsilateral, contra = contralateral, Diff = difference, NR = no response, - = not done. Normal adult values derived from reference 3.

Supplementary Table 3. Markers used to define the scRNAseq cell clusters

Cluster #	Cluster Identification	Cluster Marker, reference
1	r3-r7 mitotic progenitors (rhombomere 3 – rhombomere 7 mitotic progenitors)	*Rest*[4]<br>*Id3*[5]<br>*Hoxb1*[6]<br>*Isl1*[7]<br>*Nr2f1*[8]<br>*Nr2f2* (this study)<br>*Top2a*[9]
2	Mitotic r4MN progenitors (mitotic rhombomere 4-derived motor neuron progenitors)	*Hoxb1*[6]<br>*Isl1*[7]<br>*Nr2f1*[8]<br>*Nr2f2* (this study)<br>*Top2a*[9]
3	r4MN precursors (rhombomere 4-derived motor neuron precursors)	*Hoxb1*[6]<br>*Isl1*[7]<br>*Nr2f1*[8]<br>*Neurod4*[10]<br>*Mybpc1* (this study)
4	Biopotent bipotent r4MNs (biopotent rhombomere 4-derived motor neurons)	*Isl1*[7]<br>*Nr2f1*[8]<br>*Nr2f2* (this study)
5	IEEs (inner ear efferent neurons)	*Gata2*[11]<br>*Gata3*[12]<br>*Gad2* (this study)<br>*Chm2* (this study)<br>*Il1rap* (this study)<br>*Gpr149* (this study)
6	FBMNs (facial branchial motor neurons)	*Lingo2*[13]<br>*Tmeff2*[14]<br>*Alk* (this study)<br>*Shox2*[15]<br>*Cdh8*[16]<br>*Slc44a5* (this study)<br>*Cbln2* (this study)<br>*Zfpm2*[17]<br>*Syt4* (this study)
7	CN V, CN X, CN XII precursors (trigeminal, ambiguous, hypoglossal nuclei)	*Hoxa3*[18]
8	CN V, CN X, CN XII (trigeminal, ambiguous, hypoglossal nuclei)	*Hoxa3*[18]<br>*Dgkb* (this study)<br>*Kcnip4* (this study)<br>*Blc2* (this study)<br>*Kcna5* (this study)<br>*Runx1*[19]
9	FVMNs (facial visceral motor neurons)	*Mgat4c* (this study)<br>*Irx1* (this study)<br>*Irx2* (this study)
10	CN V (trigeminal motor nucleus)	*Sema3d* (this study)<br>*Sox1* (this study)
11	CN VI (abducens)	*Mnx1*[20]<br>*Rasgef1c* (this study)<br>*Isl2*[21]
12,13	Vestibuloacoustic ganglion precursors and ganglion	*Neurod1*[22]<br>*Sct* (this study)<br>*Tlx3*[22]<br>*Tlx2*[22]<br>*Six1*[23]
14	Dorsal hindbrain interneurons	*Nrn1* (this study)<br>*Slc17a6*[24]<br>*Prrxl1*[25]<br>*Lbx1*[26]
15	Neural crest	*Gpc3* (this study)<br>*Twist1*[27]
16	Hair cell precursors?	*Krt8*[28]

		Clnd7[28]<br>*Epcam*[29]
n/a	n/a	*Dnajb8*[30]

Supplementary Table 4. Primer and Oligo Sequences

	Forward Primer	Reverse Primer
**Screening (Sanger)**		
Chr3 cRE2 conserved region	GGCAACAGGAAGGAAGCAGA	CTGCATCTTGAGGCTCGGG
**Screening (ddPCR)**		
DNAJB8 probe: CTGTCCCAGAACTCAA	ACCGGCTACACCTTCCGTAAC	CCACCACGGTCACTATTGAATG
Chr3 conserved region probe: AGCCACAGCTGGCCA	CTGTGCTAAGCAGACCCACTTG	GCCCCCACTACAGCATTAACC
hTERT	TaqMan™ Copy Number Reference Assay, human, TERT	Cat# 4403316
RNaseP	TaqMan™ Copy Number Reference Assay, human, RNase P	Cat# 4403326
**Breakpoint spanning PCR primers**		
Fam1	GCTCAGTCTGAAGCCCATTG	TCTCCCTGGGATGTTCTGTC
Fam2	TGCAAACAGAGGGAAGAAGG	GGCCTGTTTCTCACAAGGAG
Fam10	TCCTCAGTTACTCGAAGCCTC	GAAGAGTGGCTAGGAGGTCAG
Fam11	GAAGAGTGGCTAGGAGGTCAG	ATGGAATGTTGTGCTGGTCC
Fam12	CTGGCCTTTAAGTCCTGAGC	GTGCTGTGGGGCTTGTATTC
**Haplotyping primers**		
**Fam2**		
Chr3:128246197, 128246228	TCCCAATGAGAGATGGCCTA	GGGGTGATAAGAGGGGTGAT
Chr3:128251841, 128251885	GGTCTTGGTCCAAATACCACA	CACCACCATGCTTGGCTAAT
Chr3:128253165, 128253309, 128253509	GGAGCCTACACTTCAGACCAA	AAGAGCTTGAGGGGGAGGT
Chr3:128257374, 128257544	TGCAATGCCTAGAAGACCTG	TGACTTGTTGGGTAGGTGGT
Chr3:128316909, 128316965	ACCAACCGGTGACAGATGTT	AGCTGACTGGGGATCTGTGA
Chr3:128055511	CCATGTCTCCAACCTGCTG	TGTGGGTAGTCAGGGAGGAC
Chr3:128168598	CACCCACCTATGCAGGTTCT	ATCAGACTGGGATGGGAGTG
**Fam7 and Fam8**		
chr3:127256437	CCTGAGGGTTGGGAATGGAA	GCATGACCACAGGAACGATG

chr3:127291274	AGTTGGCTGTGTCCTGGATG	AGCACTGGGATCATAAGGCA
chr3:127514425	CATGTTCTCACAAGTGGGAGC	TTGTGCAATGACCCATATTCCT
chr3:127881362	CTGGAGTTGCTTGCCTTCAC	TTGCCTCAAGAAGCCAGAGT
chr3:127942573	CCTGAACTGGTAGCTGGTAGC	AGGGATAAGCCAACCTATTT
chr3:128034859,<br>128034860	GAAGTCCCTGCACACTGTCA	TGAATAAATACCACGCTCTTTGA
chr3:128185191	AAAGCTTCTGTTGGGCCATG	CCTCCTATCCCATCACCACC
chr3:128191414	GCCTCCACTCTCCTAACTCC	GTTACTGGCAGAGCTCACCT
chr3:128205486	AAGATCAGGGTAGGCAGAGC	ACGAGGTGGACGTCTTCTTC
chr3:128510561	ATTCACCTTCCTTGGCCTCC	AGTGGCAGGTCATCAGCAG
chr3:128739889	CTGAGATCACGCCACTGCAT	CCACCCTTCCAGTCACTGAG
chr3:129072890	AGGGAGTGTGGAGAGTCAGG	TCGAAAGCACTCCAAGGTCA
**In situ probes (**T7 RNA polymerase recognition sequence bolded)		
Dnajb8	GGGGACTGGACCCTTTTTCC	**GCGTAATACGACTCACTATAGGG**<br>TCGCTTGGTGGTCACCTTAC
Gata2	CCAGGATGGGTGGAACATAC	**GCGTAATACGACTCACTATAGGG**<br>GACCCAAGAACCACTCAAA
Isl1	CACTGTGGACATTACTCCCTC	**GCGCTAATACGACTCACTATAGG**<br>**G**AACATCTGAATGAATGTTCCTCA<br>TGCC
**EMSA probes**		
pWT	ACAGAGGTCAGGTGGTTAATGCTG<br>TAGTGGGGGCTTTGATGAAGTTAG<br>TC	GACTAACTTCATCAAAGCCCCCAC<br>TACAGCATTAACCACCTGACCTCT<br>GT
p3	ACAGACGTCAGGTGGTTAATGCTG<br>TAGTGGGGGCTTTGATGAAGTTAG<br>TC	GACTAACTTCATCAAAGCCCCCAC<br>TACAGCATTAACCACCTGACCTCT<br>GT
p4	ACAGAGATCAGGTGGTTAATGCTG<br>TAGTGGGGGCTTTGATGAAGTTAG<br>TC	GACTAACTTCATCAAAGCCCCCAC<br>TACAGCATTAACCACCTGATCTCT<br>GT
p5	ACAGAGGCCAGGTGGTTAATGCTG<br>TAGTGGGGGCTTTGATGAAGTTAG<br>TC	GACTAACTTCATCAAAGCCCCCAC<br>TACAGCATTAACCACCTGGCCTCT<br>GT
p7-8	ACAGAGGTCAGGTGGTTAATGCTG<br>TAGTGGGGGCTTTGATGAGGTTAG<br>TC	GACTAACCTCATCAAAGCCCCCA<br>CTACAGCATTAACCACCTGACCTC<br>TGT

p9	ACAGAGGTCAGGTGGTTAATGCTG TAGTGGGGGCTTTGATGAAATTAG TC	GACTAATTTCATCAAAGCCCCCAC TACAGCATTAACCACCTGACCTCT GT
p5-p9	ACAGAGATCAGGTGGTTAATGCTG TAGTGGGGGCTTTGATGAAATTAG TC	GACTAATTTCATCAAAGCCCCCAC TACAGCATTAACCACCTGGCCTCT GT
**LacZ mutagenesis and sequencing primers**		
cRE1	ATGCTTGCTGGGATAAGTTAGAGG	AGCTTTGTGGAACACTCAGGAGT
cRE2	CAGGCTTTCACAGAATCCTTCTGG	CTGCCTTTAAAATGGTGGTGATGA
cRE3	AGAGACAAAGGACCTAGTATGAGA	GATGTACCTGGGTTGGGATGG
cRE2*A site-directed mutagenesis primer	CAGCATTAACCACCTGGTGTCTGT CCC	GGGACAGACACCAGGTGGTTAAT GCTG
cRE2*B site-directed mutagenesis primer	CCTGGGACTAATCTAATCAAAGCC CCC	GGGGGCTTTGATTAGATTAGTCC CAGG
***Isl*^{MN}*-GFP* genotyping**		
	TATATCATGGCCGACAAGCA	TCAGGAGAGCACACACTTGC
***Gata2*$^{KO/flox}$ genotyping**		
Gata2 flox allele	CCAGGATGGGTGGAACATAC	GAAGGACCCCAAGAACACAA
Gata2 KO allele	TGCAACTGGAGACAGCAACT	GAAGGACCCCAAGAACACAA
***Gata3*$^{TLZ/flox}$ genotyping**		
Gata3 wildtype allele	CAGGAGTCCGCGGACCTCC	CGTTGAGGACCGCGGGGTG
Gata3 TLZ allele	CAGGAGTCCGCGGACCTC C	TGCCTTTACTGACCATGCGAG
Gata3 flox allele	GTGCAGCAGAGCAGGAAACTCTCA C	CAGTCTCTGGTATTGATCTGCTTC TT
***Phox2bCre* genotyping**		
	CACCGTCTCCACATCCATC	CGGTTATTCAACTTGCACCA
***Fam5*^{SNV} mouse genotyping**		
Taqman assay, ThermoFisher	Assay ID = AN7DWV6	Order assay using the assay ID
***cRE1*^{dup} mouse genotyping**		
For *cRE1*^{dup} wild type allele	ACCCTTTCCTCTGACCCTGT	CCTGCCGAGGAAAGAGGCTG
For *cRE1*^{dup} mutant allele	GTGAGGGGTGGAGATGGAG	GGGGAGGTTGTGCAGTAGG

Supplementary Methods

1. Genetics

DNA source. Genomic DNA was extracted from collected samples using Qiagen Puregene blood kits (Qiagen, Hilden, Germany) or Oragene purifier solution (DNA Genotek, Ontario, Canada).

SNP CNV analysis. For SNP CNV discovery, Log R Ratio (LRR) and B Allele Frequency (BAF) along with other SNP information were generated by Genome Studio v2.0 (Illumina) for CNV analysis. PennCNV v1.05[31] and QuantiSNP v2.3[32] were used to generate CNV calls respectively. The two call sets were merged where CNVs called by both methods in the same individual were merged if the size of their overlap was >30% the size of their union. The merged set of CNVs was further filtered with the following criteria: a) segregation with disease status; b) location in the linkage regions and c) overlapping or proximate candidate genes. The LRR and BAF signals of CNVs passing the filtering criteria were visualized using in-house R scripts and manually checked.

Exome Sequencing. For exome sequencing, DNA libraries were prepared using Nimblegen SeqCap EZ Exome v2 kits for all individuals in Fam9 and individuals III-1, IV-1, IV-2, IV-5, and IV-6 in Fam1 and were sequenced on an Illumina Hiseq 2500. Library preparations were performed at the National Human Genome Research Institute, and sequencing was performed at the NIH Intramural Sequencing Center (NIH/NISC, Comparative Sequencing Program). The remaining DNA libraries from Fam1 (III-2, III-3, III-4, IV-3, IV-4) were prepared using Agilent SureSelect Human All Exon v4 kit and sequenced on an Illumina HiSeq 2000. Libraries were generated in the Engle lab and were sequenced at the HMS OGI. All samples were well covered with at least 98% of the exomic region showing 10x coverage.

Whole genome sequencing. For cohort 1, WGS was performed using the following methodology: Library preparation using the KAPA Hyper PCR-free kit (KK8505, KAPA Biosystems Inc.) and sequencing using Reagent Kit v2.5 on an Illumina HiSeq X platform were performed at Baylor College of Medicine. Raw 30X PCR-free WGS data were reprocessed at the Broad Institute. Raw read data were realigned to the GRCh37/hg19 human reference sequence using BWA v0.78 and reprocessed using the Broad's Picard Toolkit v1.141. The resulting BAM files were then subjected to variant calling using the Genome Analysis Toolkit (GATK 4.0 HaplotypeCaller)[33]. In the final step of variant calling, each site in the genome was jointly genotyped alongside a collection of over 20,000 reference genomes assembled by the Broad Institute to improve variant calling accuracy and harmonize data across samples. Variant filtering was performed with GATK's Variant Quality Score Recalibrator (v3.5-0-g36282e4) and custom hard filters as required. LUMPY v0.2.13[34] and Manta v1.1.0[35] were applied to the generated BAM files to call CNVs. At each stage in the variant-calling process, QC metrics were assessed to identify problems with DNA or sequencing quality. Sequence quality metrics (such as uniformity of coverage, transition/transversion ratio, indel length profiles) were compared with an internal database of reference genomes, and outlier samples were flagged for deeper investigation. Heterozygosity of common variants on chrX and coverage of sites on chrY was used to confirm gender and to identify sex chromosome aneuploidy. Variant calls from 12,000 well-covered variant sites were used along with a large reference panel to infer geographical ancestry of samples by principal components analysis, and to infer pairwise relatedness of the samples, to determine unexpected duplicates, cryptic relatedness, or unexpected patterns of relatedness within reported families.

For cohort 2, WGS was performed at BGI (BGI, Hong Kong, China) on a BGISeq500 sequencing platform for Fam10 and Fam14 and at the Radboudumc (Nijmegen, the Netherlands) on a Novaseq6000 (Illumina) sequencing platform for Fam11, Fam12 and Fam13, following manufacturers recommendations. Sequencing used a paired-end module of 2x100 base pairs on BGISeq500 and paired-end module of 2x150 base pairs on Novaseq6000, both with minimal median coverage of 30-fold per genome. BWA v0.78[36] was used to map the reads to the GRCh37/hg19 reference genome build, and bam quality control was performed with Qualimap v2.2.1[37]. Data quality is ensured by checking several quality metrics, including insert size,

percentage mapped reads, percentage duplicated mapped reads, coverage, percentage of bases with more than 20-fold coverage and error rate. Alignment files entered several variant calling pipelines. Calling of SNVs and small indels was performed with xAtlas v0.1[38]. Variants were subsequently annotated with a proprietary Radboudumc inhouse developed annotation pipeline, that uses the Variant Effect Predictor[39] (VEP v109) and Gencode v34lift37 basic gene annotations. In addition, information on allele frequency in the general population was added from GnomAD v2.1.1 and from a Radboudumc inhouse database. Additional annotations included CADD score v1.6, SpliceAI, OMIM and KEGG pathways. SNVs and small indels were additionally analyzed with Exomizer v13.0.0 using ReMM v0.3.1[40]. CNVs were identified with Control-FREEC v11.6[41] and Canvas Copy Number Variant Caller v1.40.0 [42], both using read depth for the detection of copy number changes. Calling of structural variants (SVs) was performed with the Manta v1.1.0[35], which uses a paired-end and split read evidence approach. Both CNVs and SVs were annotated with a proprietary Radboudumc inhouse developed pipeline, based on ANNOVAR (**2022**Aug02)[43] and Gencode v34lift37 basic gene annotations[44], with additional information on population allele frequency from GnomAD v2.1[45],1000G v8 [46] and GoNL SV database [47]. Finally, all the prioritized SNVs and CNVs were visually confirmed by Integrative Genomics Viewer (IGV v2.8.0)[48].

Whole genome sequence interpretation. The duplication on chromosome 3 linkage region in Fam1 identified at the discovery stage was confirmed by the CNV results from both LUMPY v0.2.13 and Manta v1.1.0. Both CNV call sets were also screened against the chromosome 3 linkage region in order to identify more CNVs segregating in additional families. The DNA sequences at the CNV breakpoints were examined for microhomology with Clustal Omega (http://www.ebi.ac.uk/Tools/msa/clustalo/; European Bioinformatics Institute).

Whole genome sequences from families with HCFP were analyzed for SNVs and small indels in the previously identified overlapping duplication interval of chr3:128,174,924-128,192,750 using seqr v1.0 (https://seqr.broadinstitute.org/), a variant analysis tool developed at the Broad Institute, with a custom inheritance pattern in order to include variants with incomplete penetrance. Identified variants were evaluated for frequency in the Genome Aggregation Database (gnomAD)[45], and for cross-species conservation and functional significance, including DNase hypersensitivity, TF binding, and histone acetylation using ENCODE[49] and other data through the UCSC genome browser.

Variant validation, co-segregation, and haplotypes. SNV confirmation and segregation was evaluated in all available family members by Sanger sequencing. For any pedigree in which a *de novo* variant was identified, family relationships were verified by analysis of 24 microsatellites using the PowerPlex Fusion System (Promega). In Fam2, Fam7, and Fam8, rare variants (MAF<10%) identified by WGS that segregated with disease status were amplified and Sanger sequenced using primers in Supplementary Table 4. To evaluate relatedness between Fam7 and Fam8, PCR primers were made to rare variants identified in Fam7 WGS based on gnomAD frequencies and sequenced in Fam8 (Supplementary Table 4). For Fam9 and Fam14, rare variants were compared directly from their WGS.

The identified duplications in Fam1,2,10-12 were validated by breakpoint-spanning PCR and/or Sanger sequencing. Co-segregation analysis of Fam1 and 2 was performed using ddPCR. Co-segregation analysis of Fam10-12 was performed with agarose gel electrophoresis to confirm the presence or absence of a PCR product of the expected size for each participant.

2. Clinical Testing

A subset of participants from Fam1, Fam3, and Fam9 in Cohort 1 received extensive clinical testing at the NIH Clinical Center based on the following protocols:

Neuropsychological testing. Clinical psychologists supervised assessments that included age-appropriate instruments to evaluate cognitive or adaptive functioning and screen for concerns in neurodevelopmental and neuropsychological domains.

Magnetic resonance imaging (MRI). 3.0T Philips Achieva MRI sequences include the following: 1) 1 mm sagittal 3D T1 turbo field echo (TFE) sensitivity encoding (SENSE); 2) 3 mm axial fluid attenuated inversion recovery (FLAIR); 3) 3 mm axial T2 constant level appearance (CLEAR), for brain and face/muscles of mastication; 4) 3 mm coronal short tau inversion recovery (STIR) olfactory bulbs, modified to extend through the pituitary; 5) 2 mm coronal T1 and T2 orbits, modified to extend from the mid-globe through the back of the sella to allow better imaging of the extraocular muscles and optic nerves; 6) 3D gradient echo balanced fast field echo (BFFE) of the brainstem for imaging of most of the cranial nerves; 7) 1mm 3D volume isotropic turbo spin echo acquisition (VISTA) for imaging of the facial nerve cisternal segment and within the internal auditory canal.

Audiology testing. Audiometric evaluations consisted of ear-specific air conduction (250-8000 Hz) and bone conduction (250-4000 Hz) pure-tone thresholds at octave frequencies. All behavioral pure-tone threshold assessments were conducted using clinical audiometers (GSI-61, Grason-Stadler) in double-walled sound booths that met American National Standards Institute criteria (ANSI, 2003, 2004). Normal hearing was classified as audiometric thresholds <=25 dB HL. Middle ear function was evaluated using a GSI Tymp Star (GSI-33; Grason-Stadler) immittance bridge with a 226-Hz probe tone. Acoustic stapedial reflex thresholds were evaluated using published normative data[50].

Neurodiagnostic auditory brainstem responses (ABR) were performed using an Audera evoked potential system (Grason-Stadler). Responses were obtained by averaging single polarity condensation and rarefaction broadband clicks presented at 95 or 85 and 0 dB nHL at a rate of 8.3 clicks per second using an Fz to ipsilateral and contralateral earlobe electrode montage and a grounding electrode placed at Fpz. Absolute and interpeak latencies for waves I, III and V of the ABR were evaluated using normative data[51].

Neurophysiology testing. The electrophysiology studies were performed using a Viking Select EMG machine (Natus, Middleton, WI)[52]. Subjects underwent blink studies and cranial nerve 7 (CN7) motor nerve conduction studies (MNCS) using standard techniques. Blink studies were performed by stimulating at the supraorbital notch with bilateral periorbital recording sites. The CN7 MNCS were recorded in the upper face from the orbicularis oculi muscle (CN7-OOC), and in the lower face from the orbicularis oris muscle (CN7-OOR). Optional studies per protocol included median and fibular MNCS; median and sural sensory nerve conduction studies (SNCS); and needle EMG (NEMG) to face (orbicularis oculi, mentalis) and limbs (biceps brachii, tibialis anterior). The needle EMG was limited to adults who agreed to it and was not in children. Published normal amplitudes for CN7 MNC OOC are ≥ 1.0 mV though not stated for CN7 MNC OOR and for the reflexes are: R1 ≤ 13 ms, ipsilateral R2 ≤ 41 ms, and contralateral R2 y ≤ 44 ms[3].

3. Electrophoretic mobility shift assays

5'-IRDye 700-labeled HPLC-purified oligonucleotides (IDT) and unlabeled competitors were annealed to generate double-stranded DNA probes for the WT and mutant chromosome 3 sequence of interest (Supplementary Table 4). The binding reaction was performed in binding buffer containing 10 mM Tris, 50 mM KCl, 3.5 mM DTT, 50 µg/µl poly(dI•dC), 0.025% Tween®20. Labeled probes were incubated with and without HeLaScribe® nuclear extract, Gel shift assay grade (#E352A, Promega) or HEK293T cells nuclear extract (ATCC CRL-3216) transfected with *NR2F1* plasmid (GeneCopeia, EX-A1000-M02). Nuclear extracts from the HEK293T cells were prepared using NE-PER reagents (#78833, Thermo Fisher Scientific) with the addition of protease inhibitor cocktail (#11836153001, Millipore-Sigma) and assayed for total protein using the Pierce BCA protein assay kit (#23225, Thermo Fisher Scientific). Presence of the recombinant NR2F1 protein in the nuclear extract was evaluated by Western blot using anti-NR2F1 antibody (D4H2 Rabbit mAb #6364, Cell Signaling Technology, Danvers, MA). After pre-incubating 5 ug nuclear extract and variable excess unlabeled competitor in the binding buffer for 5 minutes, 50 fmol labeled oligo was added and incubated 25 minutes at RT in the dark. For the supershift assay, 1 µg of anti-NR2F1 antibody from either Cell Signaling (D4H2 Rabbit mAb #6364) or Perseus Proteomics (#PP-H8124-00, mouse mAb) and respective isotype controls (WNT3A Rabbit mAb, Cell Signaling, #2721; anti-HA, IgG2a mouse monoclonal, Thermo Fisher Scientific, #5B1D10) were added during the 5' pre-incubation step. Samples were mixed with orange loading dye (#927-10100, LI-COR Biosciences) and loaded on a 4-20% Novex TBE polyacrylamide

gel (Thermo Fisher Scientific) that had been pre-run in TBE buffer. Gels were visualized using an Odyssey imaging system (LI-COR Biosciences) and data analyzed using ImageStudio Software v5.2 (LI-COR bioscience).

4. *LacZ* Assay

Transgenic E11.5 mouse embryos were generated as described previously[53]. Briefly, super-ovulating female FVB mice were mated with FVB males and fertilized embryos were collected from the oviducts. Regulatory elements were amplified from human genomic DNA and their allelic versions were created using sets of complementary primers with desired variants (Supplementary Table 4). Inserts generated in this way were cloned into donor plasmid containing minimal Shh promoter, *lacZ* reporter gene and H11 locus homology arms (Addgene, 139098) using NEBuilder HiFi DNA Assembly Mix (New England Biolabs, Ipswich, MA, E2621). The sequence identity of donor plasmids was verified using short-read sequencing. Plasmids are available upon request. A mixture of Cas9 protein (Alt-R SpCas9 Nuclease V3, IDT, Coralville, IA, 1081058, final concentration 20 ng/μL), hybridized sgRNA against H11 locus (Alt-R CRISPR-Cas9 tracrRNA, IDT, 1072532 and Alt-R CRISPR-Cas9 locus targeting crRNA, gctgatggaacaggtaacaa, total final concentration 50 ng/μL) and donor plasmid (12.5 ng/μL) was injected into the pronucleus of donor FVB embryos. The efficiency of targeting and the gRNA selection process is as previously described[53]. Embryos were cultured in M16 with amino acids at 37°C, 5% CO_2 for 2 hours and implanted into pseudopregnant CD-1 mice and collected at E11.5 for *lacZ* staining as described previously[53]. Briefly, embryos were dissected from the uterine horns, washed in cold PBS, fixed in 4% PFA for 30 min and washed three times in embryo wash buffer (2 mM $MgCl_2$, 0.02% NP-40, and 0.01% deoxycholate in PBS at pH 7.3). They were subsequently stained overnight at room temperature in X-gal stain (4 mM potassium ferricyanide, 4 mM potassium ferrocyanide, 1 mg/mL X-gal and 20 mM Tris pH 7.5 in embryo wash buffer). PCR using genomic DNA extracted from embryonic sacs digested with DirectPCR Lysis Reagent (Viagen Bioltech, Los Angeles, CA, 301-C) containing Proteinase K (final concentration 6 U/mL) was used to confirm integration at the H11 locus and test for presence of tandem insertions (refer to Osterwalder 2022 for details[53]). Only embryos with donor plasmid insertion at H11 were used. The stained transgenic embryos were washed three times in PBS and imaged from both sides using a Leica MZ16 microscope and Leica DFC420 digital camera (Extended Data Fig. 3 and in the Vista enhancer browser[54] (https://enhancer.lbl.gov/) with the following hs numbers: hs2664 (cRE1), hs2665 (cRE2), hs2666 (cRE3), hs2667 (cRE1+cRE2), and hs2668 (cRE2+cRE3)).

5. Mouse Lines

***Gata2^{flox}* mouse line** was generated by Stuart Orkin's lab on a BALBc/C57Bl6 (Jackson Labs, 00661 and 000664) background. Homologous recombination was used to place loxP sites flanking exons 5 and 6 of *Gata2*, allowing the conditional deletion of the C-terminal zinc finger DNA binding domain that is required for function. To generate a constitutive *Gata2* knockout allele, *Gata2$^{flox/flox}$* mice were bred with the *Ella-Cre* line (Jackson Labs,003724, B6.FVB-Tg(EIIa-cre)C5379Lmgd/J), and *Gata2$^{flox/+}$*; *Ella-Cre^{+}* progeny were back crossed twice to the *Ella-Cre* line to eliminate mosaicism and ensure germline deletion. To generate experimental litters containing *Gata2* cKO mice, *Gata2$^{KO/+}$* breeders were crossed to the BAC transgenic Phox2b-Cre line (Jackson Labs 016223, B6(Cg)-T(Phox2b-Cre)3Jke/J) that drives expression in cranial motor neurons, generating *Gata2$^{KO/+}$*;Phox2b-Cre^{+} male breeders. These males were crossed to *Gata2$^{flox/flox}$* females to generate experimental *Gata2$^{KO/flox}$*;Phox2b-Cre^{+} conditional *Gata2* knockouts which were viable into adulthood.

The *Gata3^{taulacZ}* mouse line was a gift of Frank G. Grosveld (Erasmus Medical Center)[55]. The mice were generated with homologous recombination using a targeting vector containing an expression cassette for tau-tagged *lacZ* inserted at the *Gata3* start codon and were received and maintained on a mixed 129/C57BL6/CD1 background.

The *Gata3$^{flox/+}$* mouse line was a gift of Jinfang Zhu (NIH/NIAID)[56]. The mice were engineered with exon 4 flanked by LoxP sites using homologous recombination and were received and maintained on a mixed 129/C57BL6/CD1 background.

***Gata2*$^{KO/flox}$; Phox2bCre^{+} cKO** mice were generated by crossing *Gata3*$^{tlz/+}$ mice Phox2b-Cre^{+} mice to generate *Gata3*$^{tlz/+}$;Phox2b-Cre^{+} male breeders. Crossing the *Gata3*$^{tlz/+}$;Phox2b-Cre^{+} males to *Gata3*$^{flox/flox}$ females generated litters containing experimental *Gata3*$^{tlz/flox}$;Phox2b-Cre^{+} cKO mice which were viable through adulthood.

The *Fam5*^{snv} mouse line harboring the mm10:chr6:88224892A>G SNV was generated in the Kirby Neurobiology Gene Editing Core at Boston Children's Hospital by CRISPR-Cas9[57]. These mice were maintained on a mixed BALBc/C57Bl6 background. For scCUT&Tag experiments, *Fam5*$^{snv/snv}$ mice were crossed onto the motor neuron-specific Isl1^{MN}-GFP reporter background (Jackson Labs 017952, STOCK Tg(Isl1-EGFP*)1Slp/J) and intercrossed to generate Isl1^{MN}-GFP;*Fam5*$^{snv/snv}$ breeder mice. These mice were intercrossed to generate litters of E10.5 Isl1^{MN}-GFP;*Fam5*$^{snv/snv}$ used for scCUT&Tag. The variant and surrounding sequence was confirmed by Sanger sequencing.

The *cRE1*$^{dup/+}$ mouse was generated at the Gene Targeting and Transgenic Facility at Albert Einstein College of Medicine by CRISPR-Cas9. The cRE1dup Homologous Recombination Donor (HRD) plasmid generated with the low-copy plasmid vector (pSMART-LC-AMP) contained two copies of human cRE1 sequence (hg19:chr3:128,175,708-128,176,563) with 2101 bp 5' and 4215 bp 3' homologous arms. A single gRNA (98/58; GAGGCTGGAACAGAACCGAA; mm10:chr6:88225941-88225960) targeting downstream of *GATA2* was used to direct the insertion between mouse cRE1 and cRE2. The transgenic mouse was made on the C57Bl/6J background (Jackson Labs, 00664). To confirm the intended design, DNA isolated from a *cRE1*$^{dup/+}$ mouse was sequenced using the Pacbio HiFi protocol, sequenced on 6 SMRT cells, generating ~102Gb of HiFi read data representing 36X genome coverage with an average HiFi read length of 14kb. The reads were sequenced an average of 9 times each and processed using circular consensus sequencing (CCS), giving 99.99% accuracy. The only detected structural variant called by PacBio structural variant (pbSV) caller in the region was the intended 1729 bp insertion confirming the designed genotype. To generate timed embryonic litters for scRNAseq, *cRE1*$^{dup/+}$ males were bred with females homozygous for the motor neuron-specific Isl1^{MN}-GFP reporter (JAX:017952, CB6F1/J background).

To generate experimental embryonic *cRE1*$^{dup/+}$ litters, *cRE1*$^{dup/+}$ males were crossed to C57Bl/6J females (Jackson Labs, 00664). To generate the *cRE1*$^{dup/+}$;*Gata3*$^{tlz/flox}$;Phox2b-Cre^{+} rescue mice, *cRE1*$^{dup/+}$ mice were crossed onto a Phox2b-Cre^{+} background, and *cRE1*$^{dup/+}$;Phox2b-Cre^{+} progeny were crossed with *Gata3*$^{tlz/+}$ mice to generate *cRE1*$^{dup/+}$;*Gata3*$^{tlz/+}$;Phox2b-Cre^{+} male breeders. These males were crossed to *Gata3*$^{flox/flox}$ females to generate litters containing *cRE1*$^{dup/+}$;*Gata3*$^{tlz/flox}$,Phox2b-Cre^{+} rescue mice, which were viable into adulthood.

Genotyping primers are provided in Supplementary Table 4.

6. Single Cell CUT&Tag

Single cell CUT&Tag experiments were performed using the "Single cell CUT and Tag on 10x genomics platform" protocol from www.protocol.io (https://www.protocols.io/view/single-cell-cut-and-tag-on-10x-genomics-platform-bqbnmsme) with the modification of using the CUTANA pAG-Tn5 enzyme from Epicypher (Durham, NC, US; cat number: 15-1117) and all buffers (antibody, digitonin, digitonin-300, and tagmentation) contain 2% of BSA.

In brief, GFP-positive motor neurons lying adjacent to the r4 midline were dissected from Isl1^{MN}-GFP embryos at E10.5 (replicate 1: n = 26 WT and 8 *Fam5*$^{snv/snv}$ embryos; replicate 2: n = 26 WT and 16 *Fam5*$^{snv/snv}$ embryos) and FAC sorted based on GFP expression. Sorted cells were washed with antibody buffer (1x wash buffer, 2mM EDTA, 0.05% digitonin, 0.01% NP-40, and 2% BSA), and were incubated in anti-COUP-TF1 antibody (Millipore-Sigma, ABE1425) diluted 1:50 in antibody buffer overnight at 4°C rotating. Nuclei were washed in digitonin buffer (1x wash buffer, 0.05% digitonin, 0.01% NP-40, and 2% BSA) the next day.

Nuclei were incubated with guinea pig anti-rabbit antibody (Novus Biologicals, Centennial, CO NBP1-72763) (1:50 dilution in digitonin buffer), and then pAG-TN5 (1:20 dilution in digitonin-300 buffer). Each incubation was performed for 1 hour at room temperature on a rotator and nuclei were washed 3 times in digitonin-300

buffer (1x wash buffer, 0.05% digitonin, 0.01% NP-40, 2% BSA, and 150mM sodium chloride) in between incubations. Nuclei were then incubated in tagmentation buffer (1x wash buffer, 10mM MgCl2, 0.05% digitonin, 0.01% NP-40, 0.2% BSA, and 150mM NaCl) for 1 hour in a 37°C water bath (invert mix every 15 minutes). Stop solution was added to each sample at the end of the incubation and nuclei were washed with 1x diluted nuclei buffer (DNB) with 2% BSA and resuspended in 10-15ul of 1x DNB with 2% BSA.

Nuclei were counted and proceeded to the 10x Chromium Next GEM Single Cell ATAC v1.1 assay (10x Genomics, Pleasanton, CA) following manufacturer instructions with the following modifications: the transposition mix was prepared with 8ul of nuclei suspension, 7ul of ATAC buffer B, 56.5ul of barcoding reagent B, 1.5ul of reducing agent B, and 2ul of barcoding enzyme; 17 cycles were used at the final library amplification.

The resulting libraries were sequenced on an Illumina NextSeq500 using the NextSeq500/550 High Output v2.5 (75 cycles) kit (Illumina, San Diego, CA).

7. scCUT&Tag data analysis

Raw data obtained by Nextseq500 were preprocessed using the CellRanger-ATAC software v2.0.0 (10x Genomics). Briefly, data files were demultiplexed and converted to FASTQ using the mkfastq function. These files were then aligned to mm39 and duplicates removed. Cellranger output files (peak.bed and fragment file) were imported in Rstudio build 554 using R version 4.2.11.2. Data analysis was performed using Signac v1.5.0[58] and Seurat 4.2.0[59] packages following publicly available code and tutorials (https://stuartlab.org/signac/articles/pbmc_vignette.html), and script is available at https://zenodo.org/badge/latestdoi/637923997. Briefly, all samples (2 WT and 2 mutant replicates) were merged for joint analysis. To do this we performed several preprocessing steps: a) generated a common peak set, b) generated a fragment file and a matrix of peaks for each cell for each sample using the FeatureMatrix function; c) created a Seurat object for each sample. The combined dataset was analyzed using LSI approach for normalization and dimensionality reduction[60]. For visualization and nonlinear dimensionality reduction we used the RunUMAP function with 2:50 dimensions in the LSI space. Pseudobulk CUT&Tag plots were obtained using the CoveragePlot function for the region mm39 chr6:88,186,706-88,250,381.

8. Single Cell RNA sequencing

Cells were collected from embryonic hindbrains at E9.5 (n = 17 WT, 18 *cRE1*$^{dup/+}$ embryos from 2 litters), E10.5 (n = 12 WT, 7 *cRE1*$^{dup/+}$ embryos from 2 litters), E11.5 (n = 5 WT, 2 *cRE1*$^{dup/+}$ embryos from 1 litter), and E12.5 (n = 3 WT, 5 *cRE1*$^{dup/+}$ embryos from 1 litter).

Cell suspensions were loaded on the 10x Chromium Controller (10x Genomics, Pleasanton, CA) for Gel Bead-in-Emulsion (GEM) generation per the Single Cell 3' Reagent kits v3.1 User Guide. GEMs were subsequently submitted to reverse transcription incubation followed by cDNA amplification. Single-cell libraries were constructed from cDNA following specifications from Single Cell 3' library Kit. Average cDNA library size was measured on a TapeStation 4200 using High Sensitivity D1000 screen tape (Agilent Technologies, Santa Clara, CA) and concentration was quantified on a Qubit 2.0 Fluorometer using a High Sensitivity dsDNA assay kit (Thermo Fisher Scientific). All sample libraries were prepared as per manufacturer's instructions for paired-end sequencing on a NextSeq500 platform using a NextSeq500/550 High Output v2.5 kit (75 cycles) (Illumina, San Diego, CA).

9. Single Cell RNA-seq Analysis

The Cell Ranger v7.1 analysis toolkit from 10x Genomics was used for preprocessing of the sequencing data from our scRNA-seq samples. FASTQ files were generated using cellranger mkfastq, which were inputted into cellranger count to demultiplex cells, align reads to the mouse transcriptome build mm39, and generate the cell by gene expression matrices used in subsequent analyses. R v4.2.1 and Seurat v4.2.0[61] were used following publicly available code and tutorials (https://satijalab.org/seurat/articles/pbmc3k_tutorial.html), and script is available at https://zenodo.org/badge/latestdoi/637923997. Briefly, we calculated the percentage of reads

mapping to the mitochondrial genome, and excluded cells with more than 5% of their transcripts mapping to the mitochondrial genome and those with reads at the bottom and top one percent of the distribution for each sample. Normalization, scaling, and variable feature selection was performed using sctransform v0.2.0[62]. To limit our analysis to the developing r4 motor neuron population, we subsetted our cells to only include those expressing *Isl1* and/or *Hoxb1*. We assigned clusters using Seurat's SNN-graph approach with the first 40 principal components with a resolution of 0.4, and visualized our data using UMAP[63]. UMAP clusters were screened for defining marker genes in BBrowser v3.5.26 and provisionally identified based on previous published studies informed by the apparent paths of the contiguous pseudotime trajectories.

10. Immunohistochemistry and *in situ* hybridization

Immunostaining was performed on hindbrain cryosections from WT and *cRE1*$^{dup/+}$ embryos aged E10.5, E12.5, E14.5, and E16.5 WT with the following antibodies as previously described (REF PMID 31597102): guinea pig anti-ISL1/2 (gift from Tom Jessell, Columbia University, 1:15,000, RRID AB_2631974), rabbit anti-GATA2 (Abclonal A0677, 1:250), rat anti-GATA3 (eBioscience 14-9966-80, 1:200), rabbit anti-NR2F1 (Millipore-Sigma ABE1425, 1:500), AlexaFluor 488 anti-guinea pig (Invitrogen, A11073, 1:1000), AlexaFluor 568 anti-rabbit (Invitrogen, A11011, 1:1000), AlexaFluor 647 anti-rat (Invitrogen, A21247, 1:1000), AlexaFluor 647 anti-rabbit (Invitrogen, A-21244, 1:1000). Multichannel tissue section images were captured on a Zeiss LSM 980 confocal microscope using a 20x objective (E12.5, E14.5, E16.5) or 40x (E10.5) objective and a 3μM step size, stitched, and orthogonally projected using Zen 3.7 (Zeiss, White Plains, NY). Channel color reassignment and JPG conversion were performed using ImageJ v1.53d (NIH), and images were optimized in Adobe Photoshop 6 version 13.0x64.

For semi-automated quantification of r4MN number and birthdate, object masks were hand-drawn on each optical confocal section using the arivis Draw Objects tool to exclude from the analysis cells outside the region of interest and other identifiable nearby cranial motor neuron populations. The Blob Finder operation was used to identify potential cellular nuclei based on areas of increased ISL1 and/or GATA2 immunofluorescence. Segment feature filters were used to remove objects below the size threshold, and the Split operation bisected single objects composed of two cells. As an initial quality control step, the candidate cells were reviewed by eye to remove any objects that were not ISL1^{ON} cells (i.e. where immunofluorescent staining approximated the background level). Segment Feature Filter operations were used to further classify each ISL1^{ON} cell as either ISL1^{ON};GATA2^{OFF};EdU^{OFF} (FBMN not born during the EdU pulse), ISL1^{ON};GATA2^{ON};EdU^{OFF} (IEE not born during the EdU pulse), ISL1^{ON};GATA2^{OFF};EdU^{ON} (FBMN born during the EdU pulse), or ISL1^{ON};GATA2^{ON};EdU^{ON} (IEE born during the EdU pulse) based on mean signal intensity of GATA2 and EdU immunofluorescence, respectively. These cells were reviewed by eye as a second quality control step. Cells were called positive for EdU incorporation if approximately 90% of the associated ISL1^{ON} nucleus overlapped with EdU staining. Cells were identified as falling within the facial, VEN, or OCN nucleus FBMNs, VENs, and OCNs using hand-drawn masks based on anatomical position. To account for the varied and ectopic placement of r4 motor neurons in *cRE1*$^{dup/+}$mutants, ventral and dorsal hand-drawn masks were added. ISL1^{ON};GATA2^{ON} cells within the dorsal cells mask in caudal cryosections were identified as VENs, and ISL1^{ON};GATA2^{ON} cells within the ventral cells mask in rostral sections were identified as OCNs. Raw cell counts were multiplied by 4 to calculate the total per embryo and divided by 2 to generate counts per nucleus or side.

Riboprobe templates for in situ hybridization were generated with PCR using cDNA clone templates for Dnajb8 and Gata2 (Dharmacon), and embryonic mouse cDNA libraries (Clontech) for Isl1 (Supplementary Table 4). Brightfield images of stained cryosections were captured on an ECHO Revolve microscope using ECHO Pro version 3.7.5 (ECHO, San Diego, CA) and brightfield whole mount images were obtained on a Nikon SMZ18 dissecting microscope with NIS-Elements 5.21.03 64-bit (Nikon, Melville NY).

Supplementary References

1. Verzijl, H. T. F. M., van der Zwaag, B., Lammens, M., ten Donkelaar, H. J. & Padberg, G. W. The neuropathology of hereditary congenital facial palsy vs Möbius syndrome. *Neurology* **64**, 649–653 (2005).
2. Webb, B. D. *et al.* HOXB1 founder mutation in humans recapitulates the phenotype of Hoxb1-/- mice. *Am. J. Hum. Genet.* **91**, 171–179 (2012).
3. Preston, D. C. & Shapiro, B. E. *Electromyography and neuromuscular disorders*. (Elsevier, 2013). doi:10.1016/C2010-0-68780-3.
4. Jones, F. S. & Meech, R. Knockout of REST/NRSF shows that the protein is a potent repressor of neuronally expressed genes in non-neural tissues. *Bioessays* **21**, 372–376 (1999).
5. Ellmeier, W. & Weith, A. Expression of the helix-loop-helix gene Id3 during murine embryonic development. *Dev. Dyn.* **203**, 163–173 (1995).
6. Studer, M. *et al.* Genetic interactions between Hoxa1 and Hoxb1 reveal new roles in regulation of early hindbrain patterning. *Development* **125**, 1025–1036 (1998).
7. Song, M.-R. *et al.* T-Box transcription factor Tbx20 regulates a genetic program for cranial motor neuron cell body migration. *Development* **133**, 4945–4955 (2006).
8. Qiu, Y. *et al.* Null mutation of mCOUP-TFI results in defects in morphogenesis of the glossopharyngeal ganglion, axonal projection, and arborization. *Genes Dev.* **11**, 1925–1937 (1997).
9. Harkin, L. F. *et al.* Distinct expression patterns for type II topoisomerases IIA and IIB in the early foetal human telencephalon. *J. Anat.* **228**, 452–463 (2016).
10. Ohsawa, R., Ohtsuka, T. & Kageyama, R. Mash1 and Math3 are required for development of branchiomotor neurons and maintenance of neural progenitors. *J. Neurosci.* **25**, 5857–5865 (2005).
11. Nardelli, J., Thiesson, D., Fujiwara, Y., Tsai, F. Y. & Orkin, S. H. Expression and genetic interaction of transcription factors GATA-2 and GATA-3 during development of the mouse central nervous system. *Dev. Biol.* **210**, 305–321 (1999).
12. Karis, A. *et al.* Transcription factor GATA-3 alters pathway selection of olivocochlear neurons and affects morphogenesis of the ear. *J. Comp. Neurol.* **429**, 615–630 (2001).
13. Haines, B. P. & Rigby, P. W. J. Expression of the Lingo/LERN gene family during mouse embryogenesis. *Gene Expr. Patterns* **8**, 79–86 (2008).
14. Kanemoto, N. *et al.* Expression of TMEFF1 mRNA in the mouse central nervous system: precise examination and comparative studies of TMEFF1 and TMEFF2. *Brain Res. Mol. Brain Res.* **86**, 48–55 (2001).
15. Rosin, J. M., Kurrasch, D. M. & Cobb, J. Shox2 is required for the proper development of the facial motor nucleus and the establishment of the facial nerves. *BMC Neurosci.* **16**, 39 (2015).
16. Garel, S., Garcia-Dominguez, M. & Charnay, P. Control of the migratory pathway of facial branchiomotor neurones. *Development* **127**, 5297–5307 (2000).
17. Lu, J. R. *et al.* FOG-2, a heart- and brain-enriched cofactor for GATA transcription factors. *Mol. Cell. Biol.* **19**, 4495–4502 (1999).
18. Manzanares, M. *et al.* Independent regulation of initiation and maintenance phases of Hoxa3 expression in the vertebrate hindbrain involve auto- and cross-regulatory mechanisms. *Development* **128**, 3595–3607 (2001).
19. Theriault, F. M., Roy, P. & Stifani, S. AML1/Runx1 is important for the development of hindbrain cholinergic branchiovisceral motor neurons and selected cranial sensory neurons. *Proc Natl Acad Sci USA* **101**, 10343–10348 (2004).
20. Nugent, A. A. *et al.* Mutant α2-chimaerin signals via bidirectional ephrin pathways in Duane retraction syndrome. *J. Clin. Invest.* **127**, 1664–1682 (2017).
21. Guidato, S., Prin, F. & Guthrie, S. Somatic motoneurone specification in the hindbrain: the influence of somite-derived signals, retinoic acid and Hoxa3. *Development* **130**, 2981–2996 (2003).
22. Sun, Y. *et al.* Single-cell transcriptomic landscapes of the otic neuronal lineage at multiple early embryonic ages. *Cell Rep.* **38**, 110542 (2022).
23. Sato, S., Furuta, Y. & Kawakami, K. Regulation of continuous but complex expression pattern of Six1 during early sensory development. *Dev. Dyn.* **247**, 250–261 (2018).
24. Kohl, A., Hadas, Y., Klar, A. & Sela-Donenfeld, D. Axonal patterns and targets of dA1 interneurons in the chick hindbrain. *J. Neurosci.* **32**, 5757–5771 (2012).

25. Rebelo, S. *et al.* DRG11 immunohistochemical expression during embryonic development in the mouse. *Dev. Dyn.* **236**, 2653–2660 (2007).
26. Kohl, A., Marquardt, T., Klar, A. & Sela-Donenfeld, D. Control of axon guidance and neurotransmitter phenotype of dB1 hindbrain interneurons by Lim-HD code. *J. Neurosci.* **35**, 2596–2611 (2015).
27. Soldatov, R. *et al.* Spatiotemporal structure of cell fate decisions in murine neural crest. *Science* **364**, (2019).
28. Hartman, B. H., Durruthy-Durruthy, R., Laske, R. D., Losorelli, S. & Heller, S. Identification and characterization of mouse otic sensory lineage genes. *Front. Cell. Neurosci.* **9**, 79 (2015).
29. Roccio, M. *et al.* Molecular characterization and prospective isolation of human fetal cochlear hair cell progenitors. *Nat. Commun.* **9**, 4027 (2018).
30. Wang, F. *et al.* Dnajb8, a target gene of SOX30, is dispensable for male fertility in mice. *PeerJ* **8**, e10582 (2020).
31. Wang, K. *et al.* PennCNV: an integrated hidden Markov model designed for high-resolution copy number variation detection in whole-genome SNP genotyping data. *Genome Res.* **17**, 1665–1674 (2007).
32. Colella, S. *et al.* QuantiSNP: an Objective Bayes Hidden-Markov Model to detect and accurately map copy number variation using SNP genotyping data. *Nucleic Acids Res.* **35**, 2013–2025 (2007).
33. Poplin, R. *et al.* Scaling accurate genetic variant discovery to tens of thousands of samples. *BioRxiv* (2017) doi:10.1101/201178.
34. Layer, R. M., Chiang, C., Quinlan, A. R. & Hall, I. M. LUMPY: a probabilistic framework for structural variant discovery. *Genome Biol.* **15**, R84 (2014).
35. Chen, X. *et al.* Manta: rapid detection of structural variants and indels for germline and cancer sequencing applications. *Bioinformatics* **32**, 1220–1222 (2016).
36. Li, H. & Durbin, R. Fast and accurate short read alignment with Burrows-Wheeler transform. *Bioinformatics* **25**, 1754–1760 (2009).
37. Okonechnikov, K., Conesa, A. & García-Alcalde, F. Qualimap 2: advanced multi-sample quality control for high-throughput sequencing data. *Bioinformatics* **32**, 292–294 (2016).
38. Farek, J. *et al.* xAtlas: Scalable small variant calling across heterogeneous next-generation sequencing experiments. *BioRxiv* (2018) doi:10.1101/295071.
39. McLaren, W. *et al.* The ensembl variant effect predictor. *Genome Biol.* **17**, 122 (2016).
40. Robinson, P. N. *et al.* Improved exome prioritization of disease genes through cross-species phenotype comparison. *Genome Res.* **24**, 340–348 (2014).
41. Boeva, V. *et al.* Control-FREEC: a tool for assessing copy number and allelic content using next-generation sequencing data. *Bioinformatics* **28**, 423–425 (2012).
42. Roller, E., Ivakhno, S., Lee, S., Royce, T. & Tanner, S. Canvas: versatile and scalable detection of copy number variants. *Bioinformatics* **32**, 2375–2377 (2016).
43. Wang, K., Li, M. & Hakonarson, H. ANNOVAR: functional annotation of genetic variants from high-throughput sequencing data. *Nucleic Acids Res.* **38**, e164 (2010).
44. Frankish, A. *et al.* GENCODE reference annotation for the human and mouse genomes. *Nucleic Acids Res.* **47**, D766–D773 (2019).
45. Karczewski, K. J. *et al.* The mutational constraint spectrum quantified from variation in 141,456 humans. *Nature* **581**, 434–443 (2020).
46. 1000 Genomes Project Consortium *et al.* A global reference for human genetic variation. *Nature* **526**, 68–74 (2015).
47. Genome of the Netherlands Consortium. Whole-genome sequence variation, population structure and demographic history of the Dutch population. *Nat. Genet.* **46**, 818–825 (2014).
48. Robinson, J. T. *et al.* Integrative genomics viewer. *Nat. Biotechnol.* **29**, 24–26 (2011).
49. ENCODE Project Consortium. An integrated encyclopedia of DNA elements in the human genome. *Nature* **489**, 57–74 (2012).
50. Silman, S. & Gelfand, S. A. The relationship between magnitude of hearing loss and acoustic reflex threshold levels. *J. Speech Hear. Disord.* **46**, 312–316 (1981).
51. Schwartz, D. M., Pratt, R. E. & Schwartz, J. A. Auditory brain stem responses in preterm infants: evidence of peripheral maturity. *Ear Hear.* **10**, 14–22 (1989).
52. Lehky, T. *et al.* Differentiating Moebius syndrome and other congenital facial weakness disorders with electrodiagnostic studies. *Muscle Nerve* (2021) doi:10.1002/mus.27159.

53. Osterwalder, M. *et al.* Characterization of mammalian in vivo enhancers using mouse transgenesis and CRISPR genome editing. *Methods Mol. Biol.* **2403**, 147–186 (2022).
54. Visel, A., Minovitsky, S., Dubchak, I. & Pennacchio, L. A. VISTA Enhancer Browser--a database of tissue-specific human enhancers. *Nucleic Acids Res.* **35**, D88-92 (2007).
55. van Doorninck, J. H. *et al.* GATA-3 is involved in the development of serotonergic neurons in the caudal raphe nuclei. *J. Neurosci.* **19**, RC12 (1999).
56. Zhu, J. *et al.* Conditional deletion of Gata3 shows its essential function in T(H)1-T(H)2 responses. *Nat. Immunol.* **5**, 1157–1165 (2004).
57. Aida, T. *et al.* Cloning-free CRISPR/Cas system facilitates functional cassette knock-in in mice. *Genome Biol.* **16**, 87 (2015).
58. Stuart, T., Srivastava, A., Madad, S., Lareau, C. A. & Satija, R. Single-cell chromatin state analysis with Signac. *Nat. Methods* **18**, 1333–1341 (2021).
59. Hao, Y. *et al.* Integrated analysis of multimodal single-cell data. *Cell* **184**, 3573-3587.e29 (2021).
60. Cusanovich, D. A. *et al.* Multiplex single cell profiling of chromatin accessibility by combinatorial cellular indexing. *Science* **348**, 910–914 (2015).
61. Stuart, T. *et al.* Comprehensive Integration of Single-Cell Data. *Cell* **177**, 1888-1902.e21 (2019).
62. Hafemeister, C. & Satija, R. Normalization and variance stabilization of single-cell RNA-seq data using regularized negative binomial regression. *Genome Biol.* **20**, 296 (2019).
63. McInnes, L., Healy, J., Saul, N. & Großberger, L. UMAP: uniform manifold approximation and projection. *JOSS* **3**, 861 (2018).
