# Supplementary material for: Noncoding variants alter GATA2 expression in rhombomere 4 motor neurons and cause dominant hereditary congenital facial paresis
Source: Nat Genet. 2023 Jun 29;55(7):1149–63. doi: 10.1038/s41588-023-01424-9 (PMC10335940; doi:10.1038/s41588-023-01424-9)
Supplement: Supplementary file 2 — Reporting Summary [file 41588_2023_1424_MOESM2_ESM.pdf]

Corresponding author(s): Engle, Elizabeth C

Last updated by author(s): April 12, 2023

Reporting Summary

Nature Research wishes to improve the reproducibility of the work that we publish. This form provides structure for consistency and transparency in reporting. For further information on Nature Research policies, see our Editorial Policies and the Editorial Policy Checklist.

Statistics

For all statistical analyses, confirm that the following items are present in the figure legend, table legend, main text, or Methods section.

n/a	Confirmed	
☐	☒	The exact sample size (*n*) for each experimental group/condition, given as a discrete number and unit of measurement
☐	☒	A statement on whether measurements were taken from distinct samples or whether the same sample was measured repeatedly
☐	☒	The statistical test(s) used AND whether they are one- or two-sided<br>*Only common tests should be described solely by name; describe more complex techniques in the Methods section.*
☒	☐	A description of all covariates tested
☒	☐	A description of any assumptions or corrections, such as tests of normality and adjustment for multiple comparisons
☐	☒	A full description of the statistical parameters including central tendency (e.g. means) or other basic estimates (e.g. regression coefficient) AND variation (e.g. standard deviation) or associated estimates of uncertainty (e.g. confidence intervals)
☐	☒	For null hypothesis testing, the test statistic (e.g. *F*, *t*, *r*) with confidence intervals, effect sizes, degrees of freedom and *P* value noted<br>*Give P values as exact values whenever suitable.*
☒	☐	For Bayesian analysis, information on the choice of priors and Markov chain Monte Carlo settings
☒	☐	For hierarchical and complex designs, identification of the appropriate level for tests and full reporting of outcomes
☒	☐	Estimates of effect sizes (e.g. Cohen's *d*, Pearson's *r*), indicating how they were calculated

Our web collection on statistics for biologists contains articles on many of the points above.

Software and code

Policy information about availability of computer code

Data collection	Odyssey imaging system (LI-COR Biosciences),<br>Zen Imaging Software (2012),
Data analysis	LINKDATAGEN (2016 release),<br>MERLIN (version 0.5.4),<br>PennCNV (version 1.05, 2011Jun16),<br>QuantiSNP (version 2.3),<br>LUMPY (version 0.2.13),<br>Manta (version 1.1.0),<br>CADD score (version 1.6)<br>Rstudio b554<br>Clustal Omega (omega is version)<br>Genome Analysis Toolkit (GATK 4.0 HaplotypeCaller),<br>GATK's Variant Quality Score Recalibrator (v3.5-0-g36282e4)<br>Integrative Genomics Viewer (IGV 2.8.0),<br>seqr (version 1.0),<br>gnomAD (version 2.1 and 2.1.1)<br>Picard toolkit (version 1.141)<br>1000 genomes (version 8)<br>SpliceAI (https://spliceailookup.broadinstitute.org/)<br>BWA (version 0.78),<br>Qualimap (version 2.2.1),<br>xAtlas (version 0.1),

VEP (version 105),
Exomizer (version 13.0.0),
ReMM (version 0.3.1)
Control-Freec (version 11.6),
ANNOVAR (version 2022Aug02),
Gencode (V.34lift37)
Canvas Copy Number Variant Caller (version 1.40.0),
Genome Studio (version 2.0)
UCSC Genome Browser (hg19, mm10),
Geneious Prime (version 2021.1.1),
ImageStudio Software (version 5.2).
Cell Ranger (version 7.1) analysis toolkit (including -ATAC v2.0.0, cell ranger mkfstq, cell ranger count from10x Genomics),
R (version 4.2.1, v4.2.11.2,)
Seurat (version 4.2.0) including sctransform (version 0.2.0, Laboratory of Rajul Satijia, New York Genome Center),
Signac (version 1.5.0),
BBrowser Single Cell Browser (version 3.5.26, BioTuring),
BioVinci data visualization package (version 3.0.0, BioTuring),
ImageJ (NIH, version 1.53d),
arivis Vision4D x64,
tidyverse (version 1.3.1),
ggpubr (0.4.0 package),
rVista (version 2.0),
BD FACSDiva 8.0.2,
Adobe Photoshop 6 (version 13.0 x64)
Apple QuickTime Player (version 10.5)
Apple iMovie 10.3.5,
Microsoft 360 PowerPoint

For manuscripts utilizing custom algorithms or software that are central to the research but not yet described in published literature, software must be made available to editors and reviewers. We strongly encourage code deposition in a community repository (e.g. GitHub). See the Nature Research guidelines for submitting code & software for further information.

Data

Policy information about availability of data

All manuscripts must include a data availability statement. This statement should provide the following information, where applicable:

- Accession codes, unique identifiers, or web links for publicly available datasets
- A list of figures that have associated raw data
- A description of any restrictions on data availability

Publically available ChipSEQ datasets used in this study: GSM1817193 and GSM714811 for NR2F1; GSM714812 for NR2F2; GSM935589 for GATA2; GSM1010738 and GSM1602667 for GATA3. Conserved TFs binding sites were obtained using rVista 2.0 (https://rvista.dcode.org/). Additional epigenetic data were explored using the ENCODE database (https://www.encodeproject.org/). GRCh37/hg19 human reference genome under accession number SRA PRJNA31257 and GRCm38/mm10 mouse reference genome under accession number SRA PRJNA20689 were used for the alignment of human and mouse sequencing data, respectively. Variant frequencies were extracted from gnomAD (https://gnomad.broadinstitute.org/) and 1000G (https://www.internationalgenome.org). Common structural variants data were obtained by DGV (http://dgv.tcag.ca/dgv/app/home) and from the GoNL SV database (https://www.nlgenome.nl/login). KEGG PATHWAY Database was also used. OMIM was queried for disease codes (omim.org). Exome sequence and SNP data from a subset of participants are available through dbGaP Phs001383.v1.p1. WGS data from a subset of participants are available through dbGaP Phs001247.v1.p1. Single-cell RNA and CUT&Tag sequencing data are available through NCBI GEO SuperSeries record GSE223274. LacZ images are uploaded to the Vista enhancer browser (https://enhancer.lbl.gov/) and can be retrieved by their human coordinates as follows: hs2664 (cRE1) chr3:128,175,331-128,177,163; hs2665 (cRE2) chr3:128,177,164-128,179,169; hs2666 (cRE3)chr3:128,186,421-128,188,215; hs2667 (cRE1+cRE2) chr3:128,175,331-128,179,169; hs2668 (cRE2+cRE3) chr3:128,177,164-128,188,215. Mice are available on request.

Field-specific reporting

Please select the one below that is the best fit for your research. If you are not sure, read the appropriate sections before making your selection.

☒ Life sciences ☐ Behavioural & social sciences ☐ Ecological, evolutionary & environmental sciences

For a reference copy of the document with all sections, see nature.com/documents/nr-reporting-summary-flat.pdf

Life sciences study design

All studies must disclose on these points even when the disclosure is negative.

Sample size	Statistical methods were not used to determine sample size. For human genetic study, sample size was limited by number of participants with congenital facial weakness available to us and in whom we identified rare variants. For rodent cell counts and birthdating experiments, sufficient replicates were used to show reproducible EdU bioavailability over multiple independent injections and demonstrate age-dependent trends in IEE and FBMN identities and birthdates. For rodent immunohistochemistry, sufficient replicates were performed to show the reproducibility of transcription factor expression dynamically marking developing r4 MN subpopulations over developmental time. For in vitro EMSA experiments, enough replicates were performed with the WT and mutant probes in different conditions for reproducibility.
Data exclusions	Data were excluded from the study only if rendered uninterpretable for technical reasons: Damaged cryosections that precluded quantitation

Data exclusions	were excluded and a replicate sample was processed and included in the study; one E9.5 scRNAseq dataset was excluded due to high free RNA content and the experiment was repeated to generate a usable dataset; EMSA experiments were excluded if there were loading or gel-running technical problems.
Replication	All attempts at replication were successful. Rodent histology experiments were performed with a minimum of 3 biological replicates with the exception of immunostaining on E16.5 hindbrain cross sections which were performed twice. ample sizes are provided in the paper. For scRNAseq and scCUT&Tag, we conducted replicate experiments at each developmental time point from multiple pooled embryos. For EMSA, experiments were repeated multiple times in multiple days for reproducibility.
Randomization	The experiments were not randomized. Mice were allocated by genotype to wild-type or mutant categories.
Blinding	Investigators were blinded to genotypes of humans for video-based clinical interpretation of facial weakness and to mice for video-based interpretation of whisking and for cell counts and EdU quantification. Blinding was not necessary for qualitative analysis of histology.

Reporting for specific materials, systems and methods

We require information from authors about some types of materials, experimental systems and methods used in many studies. Here, indicate whether each material, system or method listed is relevant to your study. If you are not sure if a list item applies to your research, read the appropriate section before selecting a response.

Materials & experimental systems

n/a	Involved in the study
☐	☒ Antibodies
☐	☒ Eukaryotic cell lines
☒	☐ Palaeontology and archaeology
☐	☒ Animals and other organisms
☐	☒ Human research participants
☐	☒ Clinical data
☒	☐ Dual use research of concern

Methods

n/a	Involved in the study
☒	☐ ChIP-seq
☐	☒ Flow cytometry
☐	☒ MRI-based neuroimaging

Antibodies

Antibodies used

Primary antibodies:
guinea pig anti-ISL1/2 (Project ALS, RRID AB_2631974, Lot 1277),
rabbit anti-GATA2 (Abcam cat#A0677, Lot #0045400202),
rat anti-GATA3 (eBioscience cat#14-9966-80, Lot #2202643),
rabbit anti-NR2F1 (Millipore Sigma cat#ABE1425, Lot #3083591),
rabbit anti-NR2F1 (Cell Signaling Technologies D4H2 cat#6364, Lot #1)
mouse anti-NR2F1 (Perseus Proteomics #PP-H8124-00, lot number unavailable)
rabbit anti-WNT3A (Cell Signaling Technologies #2721, Lot #1)
mouse monoclonal anti-HA, IgG2a (Thermo Fisher Scientific #5B1D10)

Secondary antibodies (all from ThermoFisher Scientific):
AlexaFluor 488 anti-guinea pig (cat#A-11073, Lot#2160428),
AlexaFluor 568 anti-rabbit (cat#A-11011, Lot#2013083),
AlexaFluor 647 anti-rat (cat#A-21247, Lot#2156534),
Alexafluor 647 anti-rabbit (cat#A-37733, Lot#WL333239)

Validation

Commercially available antibodies have been validated with manufacturer-generated data and/or supporting publications found on manufacturer websites.

The specificity of the guinea pig anti-ISL1 antibody (Laboratory of Thomas Jessell and Project ALS, RRID RRID AB_2631974) was confirmed by the similarity of the sensory and motor nuclei marked by anti-ISL1 immunostaining (Figure 3,7,8; Extended Data Figures 8,9) and those marked by an Isl1 riboprobe in in situ hybridization on age-matched cryosections (Extended Data Figure 10a). This antibody was widely used by the laboratory of origin and others to mark developing motor neurons (Jung et. al. Cell 2018, PMID 29425489; Tanabe et. al Cell. 1998, PMID 9778248).

The specificity of the rabbit anti-GATA2 antibody (Abclonal A0677) was confirmed by the similarity of the IEE and hindbrain interneuron immunofluorescent staining (Figures 3,7,8; Extended Data Figure 8) and that of a Gata2 riboprobe for in situ hybridization (Extended Data Figure 10b). Specificity of the antibody was also indicated by the loss of GATA2 immunostaining from the region normally occupied by IEEs in the Gata2ko/flox; Phox2bCre+ conditional knockouts and sustained expression in the hindbrain interneurons that do not express Phox2bCre (Figure 3d). Vendor-supplied images of antibody staining on paraffin-embedded brain cross-sections confirmed specificity in epitope detection.

The specificity of the rat anti-GATA3 antibody (eBioscience 14-9966-80) was confirmed by the loss of GATA3 immunostaining from the region normally occupied by IEEs in the Gata3tlz/flox; Phox2bCre+ conditional knockouts (Figure 3e). Our GATA3 immunostaining matched published Gata3 in situ hybridization on E10.5 hindbrain cryosections (Pata et. al. Development 1999, PMID 10556076) and chromogenic detection of the β-galactosidase Gata3tlz/+ knockin reporter predicted to accurately recapitulate native GATA3 expression (Karis et. al. J. Comp. Neurol. 2001, PMID 11135239). The antibody has been shown to mark tissues known to be enriched

in GATA3, including the developing nephric duct (Sanchez-Ferras et. al., Nature Communications 2021, PMID 33976190).

The specificity of the rabbit anti-NR2F1 antibody (Millipore-Sigma ABE1425) was confirmed by published studies showing neuronal subtype-specific loss of immunofluorescent staining in NR2F1 conditional knockouts (Bovetti et. al. Development 2013, PMID 24227652; Alfano et. al. Development 2011, PMID 21965613).

For each antibody, staining of the primary and corresponding secondary antibodies was compared to that seen with secondary antibodies alone, and no confounding background staining from secondary antibodies was detected.

Eukaryotic cell lines

Policy information about cell lines

Cell line source(s)	HEK293T cells (ATCC CRL-3216). Note that HeLa cell nuclear extracts were purchased from a commercial source and were not cultured in the lab.
Authentication	HEK293T cell line was purchased from a commercial source and was not further authenticated.
Mycoplasma contamination	Cell line was tested repeatedly and tested negative for mycoplasma contamination.
Commonly misidentified lines (See ICLAC register)	None used in this study.

Animals and other organisms

Policy information about studies involving animals; ARRIVE guidelines recommended for reporting animal research

Laboratory animals	Mice were maintained in pathogen-free environments and fed ad libitum with sterile standard diet and water in a temperature, humidity, and light-controlled rooms (Boston Children's Hospital: 22°C set-point +/- 1.3°C, RH35-70% +/- 5%, 12/12 light/dark cycle, 10-15 air changes per hour; Lawrence Berkeley National Laboratory: 19-23°C, RH30-70%, 12/12 light/dark cycle, 15-20 air changes per hour; Icahn School of Medicine at Mount Sinai: 20-23°C, RH 30-70%, 12/12 light/dark, and 10-15 air changes per hour). Animals were not involved in any previous experiments. Both male and female mice aged 1-6 months were bred to generate experimental embryonic litters. Both male and female mice aged 5 weeks-5 months were assessed in the whisking assay. Age did not correlate with genotype. Fam5snv/snv mice were maintained on a 129S1/C57BL/6J mixed background. Experimental Fam5snv/snv mice were generated by intercrossing Fam5snv/snv breeders. The Gata2KO allele was generated by intercrossing Gata2flox/flox with Ella-Cre (Jackson Labs 003724) and the resulting Gata2flox/+, Ella-Cre+ mice were back-crossed to Ella-Cre two times to ensure germline deletion. Gata2KO/+ mice were crossed to Phox2bCre+ (Jackson Labs 016223) mice to generate Gata2KO/+; Phox2bCre+ breeders that, along with Gata2flox/flox mice, were maintained on a C57/Bl6 background (Jackson Labs 000664). Gata2KO/flox;Phox2bCre+ cKO embryos and control littermates were generated by crossing Gata2KO/+;Phox2bCre+ breeders to Gata2flox/flox breeders. Gata3tlz/flox and Gata3tlz/+;Phox2bCre+ mice were maintained on their 129/C57BL6/CD1 mixed background. Gata3TLZ/flox;Phox2bCre+ cKOs and control littermates were generated by crossing Gata3tlz/+;Phox2bCre+ breeders to Gata3flox/flox breeders. cRE1dup/+ mice were maintained on their C57/Bl6/BalbC mixed background and were intercrossed with Isl1MN-GFP reporter line (Jackson Labs 017952) to generate timed litters for scRNAseq and scCUT&Tag. cRE1dup/+;Gata3tlz/flox;Phox2bCre+ rescue mice and littermate controls were generated by crossing cRE1dup/+;Gata3tlz/+;Phox2bCre+ breeders to Gata3flox/flox breeders.
Wild animals	The study did not involve wild animals.
Field-collected samples	The study did not involve field-collected samples.
Ethics oversight	Institutional Animal Care and Use Committees of Boston Children's Hospital (Protocol number 00001852), the Icahn School of Medicine at Mount Sinai (Protocol number 2015-0052), and the Lawrence Berkeley National Laboratory (Protocol numbers 290003 and 290008).

Note that full information on the approval of the study protocol must also be provided in the manuscript.

Human research participants

Policy information about studies involving human research participants

Population characteristics	Male and female research participants of any age (range 6 weeks-87 years) diagnosed with isolated congenital facial weakness (some misdiagnosed with Moebius syndrome) and available family members were enrolled in the study. This was a cross-sectional observational study of subject/families enrolled over many decades through dedicated research protocols, as outlined in the Methods section. No population statistics are presented in the paper. Age, gender, genotype and phenotype information for each participant are provided in the Supplemental Clinical Data and Tables 1 and 2.
Recruitment	Subjects were referred to the research protocols in the different Institutions through their physicians, the family support group Moebius Syndrome Foundation (MSF), or self-referral. They carried a diagnosis of congenital facial weakness or Moebius syndrome. There was likely self-selection bias for subjects who have access to health care and/or interest to participate in research but this is not likely to impact results. Representation of subjects/families in research was improved through the MSF patient advocacy group. No compensation was provided to participants, but all expenses for transportation, lodging, and evaluation at the NIH Clinical Center were covered, facilitating participation of families without insurance / access to medical care.

Ethics oversight	National Institutes of Health IRB (FWA00005897) has approved the NIH Clinical Center study. Enrollment occurred under protocols approved by the Institutional Review Boards of Boston Children's Hospital, Boston, MA; CMO Radboudumc and METC East Nijmegen, Netherlands; Icahn School of Medicine at Mount Sinai, New York, NY; National Human Genome Research Institute, NIH, Bethesda, MD; American University of Beirut Medical Center, Beirut, Lebanon; Royal Victorian Eye and Ear Hospital, VIC, Australia.

Note that full information on the approval of the study protocol must also be provided in the manuscript.

Clinical data

Policy information about clinical studies
All manuscripts should comply with the ICMJE guidelines for publication of clinical research and a completed CONSORT checklist must be included with all submissions.

Clinical trial registration	NCT02055248 and NCT03059420
Study protocol	See Clinicaltrials.gov: https://www.clinicaltrials.gov/ct2/show/NCT02055248 and https://clinicaltrials.gov/ct2/show/NCT03059420
Data collection	Participants were enrolled between ~1990-present. Subjects of the NIH clinical protocol were recruited between 2014 and 2019 and data were collected at the NIH Clinical Center, Bethesda, MD. All other participants were enrolled between ~1990-present and data were collected in both clinical and research settings in Boston MA, New York City NY, Los Angeles CA, Nijmegen Netherlands, Harrow UK, Madrid Spain, Rennes France, Parana Brazil, Beirut Lebanon, and Victoria (Melbourne and Parkville), Australia.
Outcomes	Primary and secondary outcomes are provided at https://www.clinicaltrials.gov/ct2/show/NCT02055248.

Flow Cytometry

Plots

Confirm that:

☒ The axis labels state the marker and fluorochrome used (e.g. CD4-FITC).

☒ The axis scales are clearly visible. Include numbers along axes only for bottom left plot of group (a 'group' is an analysis of identical markers).

☒ All plots are contour plots with outliers or pseudocolor plots.

☒ A numerical value for number of cells or percentage (with statistics) is provided.

Methodology

Sample preparation	ISL1MN-GFP-positive and surrounding GFP negative tissues were microdissected from the hindbrain region spanning rhombomere 3 to rhombomere 7 of E9.5, E10.5, E11.5, and E12.5 mouse hindbrains. GFP-free limb buds were collected as a negative control to set GFP gating. Single cell suspensions of hindbrain tissues were generated with enzymatic digestion and trituration (Papain Dissociation System, Worthington) and passed through a 35um mesh size cell strainer by gravity to remove cell aggregates.
Instrument	BD FACSAria2
Software	BD FACSDiva 8.0.2
Cell population abundance	Isl-GFP cells comprise 2-6% of total cells sorted.
Gating strategy	GFP-positive r4-r6 neurons were purified using FACS gated to forward versus side scatter (FSC vs SSC) to exclude debris, and forward scatter width versus orward scatter (FSC-W vs FSC) followed by side scatter width versus side scatter (SSC-W vs SSC) to exclude doublets and cell aggregates. Isl1MN-GFP r4-r6 MNS were then selected based on reporter expression. Immediately prior to completion of Isl1GFP cell sorting, GFP gates were lifted to sample a representative spike of GFP-negative cells from the surrounding tissues and to reach an optimal number of total cells for the 10x Chromium scRNAseq protocol.

☒ Tick this box to confirm that a figure exemplifying the gating strategy is provided in the Supplementary Information.

Magnetic resonance imaging

Experimental design

Design type	A structural brain MRI including the internal auditory canal (IAC) and posterior fossa was offered to all participants who could cooperate with the scanning procedure without sedation.
Design specifications	N/A
Behavioral performance measures	N/A

Acquisition

Imaging type(s): Structural

Field strength: 3.0T Philips MRI scanner

Sequence & imaging parameters: Philips Achieva MRI sequences include the following: 1) 1 mm sagittal 3D T1 turbo field echo (TFE) sensitivity encoding (SENSE); 2) 3 mm axial fluid attenuated inversion recovery (FLAIR); 3) 3 mm axial T2 constant level appearance (CLEAR), for brain and face/muscles of mastication; 4) 3 mm coronal short tau inversion recovery (STIR) olfactory bulbs, modified to extend through the pituitary; 5) 2 mm coronal T1 and T2 orbits, modified to extend from the mid-globe through the back of the sella to allow better imaging of the extraocular muscles and optic nerves; 6) 3D gradient echo balanced fast field echo (BFFE) of the brainstem for imaging of most of the cranial nerves; 7) 1mm 3D volume isotropic turbo spin echo acquisition (VISTA) for imaging of the facial nerve cisternal segment and within the internal auditory canal.

Area of acquisition: Whole brain and internal auditory canal (IAC)

Diffusion MRI: ☐ Used ☒ Not used

Preprocessing

Preprocessing software: N/A

Normalization: N/A

Normalization template: N/A

Noise and artifact removal: N/A

Volume censoring: N/A

Statistical modeling & inference

Model type and settings: N/A

Effect(s) tested: N/A

Specify type of analysis: ☐ Whole brain ☐ ROI-based ☐ Both

Statistic type for inference (See Eklund et al. 2016): N/A

Correction: N/A

Models & analysis

n/a	Involved in the study
☒	☐ Functional and/or effective connectivity
☒	☐ Graph analysis
☒	☐ Multivariate modeling or predictive analysis
