# Supplementary figures and images for: Noncoding variants alter GATA2 expression in rhombomere 4 motor neurons and cause dominant hereditary congenital facial paresis
Source: Nat Genet. 2023 Jun 29;55(7):1149–63. doi: 10.1038/s41588-023-01424-9 (PMC10335940; doi:10.1038/s41588-023-01424-9)

Source Data: Unprocessed EMSA blots from Figure 4k

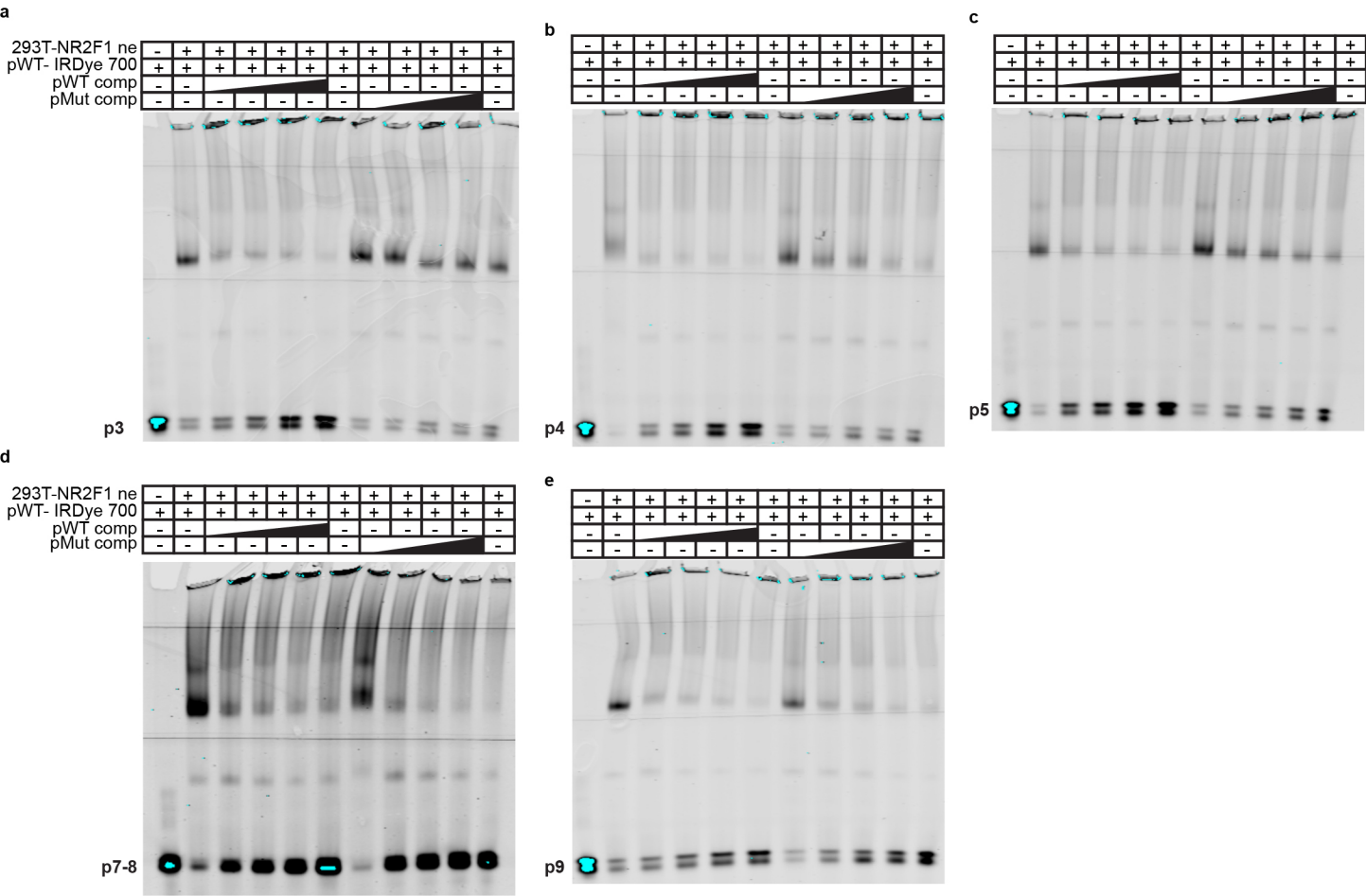

Supplement: Source Data Fig. 4 — Unprocessed EMSA blots from Fig. 4k. [file 41588_2023_1424_MOESM5_ESM.pdf]
